# Hybrid deep learning with protein language models and dual-path architecture for predicting IDP functions

**DOI:** 10.1093/bib/bbag126

**Published:** 2026-04-01

**Authors:** Jiahui Liang, Yuxian Luo, Baoquan Su, Zhenling Peng

**Affiliations:** MOE Frontiers Science Center for Nonlinear Expectations, Research Center for Mathematics and Interdisciplinary Sciences, Shandong University, 72 Binhai Road, Jimo, Qingdao 266237, China; MOE Frontiers Science Center for Nonlinear Expectations, Research Center for Mathematics and Interdisciplinary Sciences, Shandong University, 72 Binhai Road, Jimo, Qingdao 266237, China; MOE Frontiers Science Center for Nonlinear Expectations, Research Center for Mathematics and Interdisciplinary Sciences, Shandong University, 72 Binhai Road, Jimo, Qingdao 266237, China; MOE Frontiers Science Center for Nonlinear Expectations, Research Center for Mathematics and Interdisciplinary Sciences, Shandong University, 72 Binhai Road, Jimo, Qingdao 266237, China

**Keywords:** intrinsically disordered protein, deep learning, protein language models, function prediction

## Abstract

Intrinsically disordered regions (IDRs) drive essential cellular functions but resist conventional structural-function annotation due to their dynamic conformations. Current computational methods struggle with cross-dataset generalization and functional subtype discrimination. We present IDPFunNet, a hybrid deep learning model integrating convolutional neural networks, bidirectional LSTM, residual MLP, and the protein language model ProtT5 to predict six IDR functional classes: five binding subtypes and disordered flexible linkers (DFLs). Its dual-path architecture decouples binding prediction from DFL identification. Leveraging ProtT5 evolutionary embeddings, which outperformed ESM-family models and AlphaFold2 structural features (by ≥1.3% average AUC and ≥ 12.7% average APS), IDPFunNet achieves state-of-the-art performance. Across six independent benchmarks, including CAID2/3 blind tests, it consistently surpasses existing general predictors DisoFLAG and DeepDISOBind in protein-binding prediction, with AUCs of 0.866 (TE210) and 0.832 (TE83), representing significant gains of 1.5%–8.1% in AUC and 13.5%–26.7% in APS (*p*-value < 0.05), while remaining competitive with specialized DFL predictors. Further analyses show multi-task learning enhances protein/lipid/small molecule-binding (3.1%–35.1% AUC gains), BiLSTMs are optimal for DFL identification, and self-attention shows potential for nucleic acid-binding (AUC 0.831). IDPFunNet thus provides an interpretable and generalizable framework for comprehensive IDR functional mapping. The webserver of IDPFunNet is freely available at https://yanglab.qd.sdu.edu.cn/IDPFunNet/ and the standalone package can be downloaded from https://github.com/IDRIDP/IDPFunNet/tree/master.

## Introduction

Intrinsically disordered proteins (IDPs) constitute a vital class of biomolecules that perform essential cellular functions through intrinsically disordered regions (IDRs), despite lacking stable tertiary folds under physiological conditions [1–6]. These conformationally plastic regions enable multifaceted interactions with diverse binding partners—from proteins to small molecules—through adaptive folding mechanisms [7–9]. A prototypical example is the p53 transcriptional activation domain (TAD2), which binds diverse partners like S100β and CBP through conformational plasticity [10]. Beyond direct molecular recognition, IDRs frequently serve as flexible linkers (DFLs) that mediate allosteric communication between structured domains [11–13]. The p21 cyclin-dependent kinase inhibitor exemplifies this role, where its linker helix subdomain LH dynamically bridges D1 and D2 subdomains to accommodate structural variations across Cdk/cyclin complexes, thereby enabling precise cell cycle regulation [14].

The inherent structural plasticity of IDRs presents formidable challenges for high-throughput experimental characterization, necessitating computational approaches for systematic functional annotation. Early methodologies predominantly employed handcrafted features—including biophysical properties, sequence conservation patterns, and evolutionary coupling data—combined with classical machine learning algorithms (support vector machine, logistic regression, random forest) to predict IDR functions. Representative approaches from this era include ANCHOR [15], DisoRDPBind [16], MoRFchibi SYSTEM [17], DFLpred [18], APOD [19], IDPpi [20], and CLIP [21]. This paradigm evolved with deep learning advancements, yielding hybrid frameworks like flDPnn [22, 23], DeepDISOBind [24], and DisoLipPred [25] that integrate neural architectures with traditional feature engineering. The critical assessment of intrinsic disorder (CAID) experiment [26–28] systematically benchmarks these methods, revealing intriguing evolutionary trends: while ensemble machine learning models like MoRFchibi SYSTEM [17] rivaled deep learning counterparts (e.g. DeepDRPbind [29]) in CAID2 binding IDR prediction, deep neural networks dominated DFL prediction, with AlphaFold-based methods and SPOT-Disorder leading the rankings [30, 31].

Recent breakthroughs in protein language models (PLMs), including ESM-family models [32, 33] and ProtT5 [34], have further revolutionized the field. PLM applications in disorder prediction include IDP-ELM’s ensemble strategy combining ESM and ProtT5 embeddings [35], and DisoFLAG’s ProtT5-GNN hybrid achieving state-of-the-art (SOTA) performance across six functional categories [36]. Despite these continuous innovations, significant methodological challenges and knowledge gaps persist, which limit the robustness and comprehensiveness of current predictors. First, most models focus on a narrow subset of IDR functions, primarily protein-, DNA-, or RNA-binding, while other interaction modalities remain insufficiently explored [17, 22, 24]. Second, although binding IDRs and DFLs are functionally heterogeneous, they are often modeled within a unified framework [36], which may lead to task imbalance or negative transfer during joint optimization. Third, the respective contributions of PLMs, structural features, and network architectures to different IDR functional categories have not been systematically evaluated.

To directly address these limitations, we present IDPFunNet—a dual-path neural architecture specifically designed to overcome the aforementioned challenges. Our framework utilizes a hybrid of convolutional neural network (CNN) [37] and bidirectional long short-term memory (BiLSTM) [38] for comprehensive binding IDR identification, paired with a specialized BiLSTM path for DFL prediction. This design stems from the distinct biophysical signatures of these functional classes [39–41] and aims to prevent negative transfer. Through systematic benchmarking against CAID2/3 leaders and extensive ablation studies, we demonstrate IDPFunNet’s superior stability and performance in multi-category binding prediction while matching specialized DFL predictors. Furthermore, we quantify the differential impacts of AlphaFold2 (AF2) [42] structural features, multi-task learning, and self-attention mechanisms [43], thereby providing systematic, mechanistic insights into optimal model design for IDR multifunctional annotation that are currently lacking.

## Materials and methods

### Benchmark datasets

The DisProt database [44] serves as a gold standard for IDPs/IDRs, providing extensive and high-quality functional annotations such as binding IDRs and DFLs. These annotations are based on the gene ontology (GO) and the intrinsically disordered proteins ontology (IDPO). Following the GO classifications for protein-, nucleic acid-, ion-, lipid-, and other small molecule-binding, as well as IDPO terms for DFLs, we identified 1073 sequences with at least one of these six functional annotations from the DisProt database of version 9.4. To ensure annotation accuracy, we refined labels using CAID2’s [27] open-source scripts (https://github.com/BioComputingUP/caid2-reference/blob/master/src/references.ipynb), which standardize functional assignments across datasets. Given the difficulty in distinguishing RNA and DNA binding subtypes for nucleic acid binding, and to ensure adequate data for training and testing, nucleic acid binding is not divided into DNA and RNA binding subtypes.

Subsequently, we applied the program CD-HIT [45] to cluster the proteins with at least 25% sequence identity. The resulting 814 clusters were randomly divided into training, validation, and testing datasets. This division ensures the independence among these three datasets at the level of 25% sequence identity. Specifically, the training, validation, and test datasets consist of 552, 227, and 210 sequences, respectively, belonging to 463, 198, and 153 clusters. For convenience in subsequent sections, these datasets are denoted as TR552, VA227, and TE210, respectively.

To further investigate the robustness and prediction accuracy of the models, we collected 83 newly deposited sequences from the DisProt database, which were added after version 9.4 but before version 9.6. These sequences form the independent test dataset TE83. Additionally, we included four blind test datasets officially provided by the CAID2 and CAID3 competitions [27, 28], denoted as CAID2_Bind, CAID2_DFL, CAID3_Bind, and CAID3_DFL. Notably, the CAID3 datasets used in this study were obtained from the official CAID website and correspond to the version released in January 2025. The CAID2_Bind and CAID3_Bind datasets focus on generic binding events mediated by intrinsic disorder, comprising 78 and 51 proteins, respectively, each with at least one IDR binding to protein, nucleic acid, ion, lipid, and/or other small molecules. The CAID2_DFL and CAID3_DFL datasets consist of 40 and 20 sequences, respectively, where IDRs serve as flexible linkers. Notably, each of these four datasets shares less than 25% sequence identity with both our training dataset TR552 and validation dataset VA227.

In summary, the datasets TR552 and VA227 were used for training, while six independent test datasets, including TE210, TE83, CAID2_Bind, CAID2_DFL, CAID3_Bind, and CAID3_DFL, were utilized for model assessment and/or comparison. For more detailed information about these benchmark datasets, please refer to Supplementary Table 1.

### Model architecture

**Figure 1:**
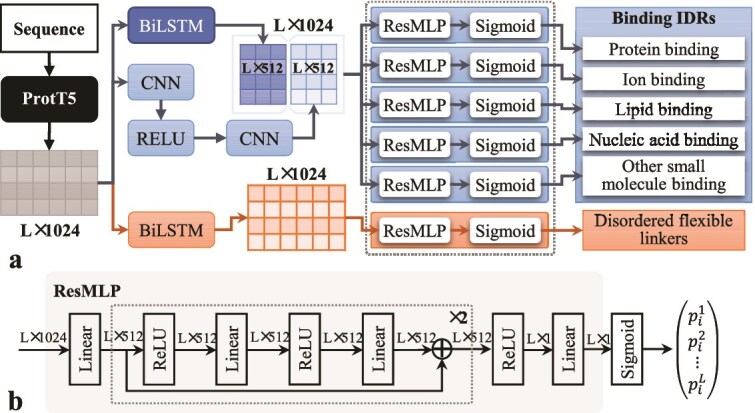
The architecture of the IDPFunNet model.

IDPFunNet represents a novel hybrid deep learning framework designed for comprehensive functional annotation of IDRs, integrating evolutionary sequence information with biophysical properties reflecting structural dynamics to concurrently predict six critical biological functions. As illustrated in Fig. 1, the architecture systematically processes input sequences through three hierarchically organized computational stages: sequence encoding, dual-path feature integration, and parallel functional prediction. This design enables simultaneous identification of five distinct interaction types (protein/nucleic acid/lipid/ion/other small molecule-binding) and DFLs, providing a unified platform for probing IDR multifunctionality.

#### Step 1: sequence encoding via ProtT5 embeddings

The framework initiates with ProtT5-based sequence embeddings, where each residue is encoded into a 1024-dimensional feature vector capturing context-sensitive evolutionary patterns and biophysical properties. Selected for its demonstrated capacity to resolve residue-level signatures [34–36], ProtT5 effectively preserves conformational plasticity signatures essential for distinguishing binding motifs from flexible linkers. This encoding establishes the foundational representation for subsequent functional discrimination.

#### Step 2: dual-path architecture for feature integration

The encoded features undergo parallel processing through two specialized pathways optimized for distinct functional categories: (i) A hybrid CNN-BiLSTM path (blue in Fig. 1a) processes binding IDR prediction. It employs a two-layer 1D CNN with a kernel size of 3 (see Supplementary Note 1) for local motif detection [37] and a single-layer BiLSTM with 512 hidden units per direction (Supplementary Note 1) for long-range dependency modeling [38]. This synergy allows the CNN to extract position-invariant binding patterns, while the BiLSTM captures context-dependent sequence correlations, each submodule outputting a 512-dimensional vector. (ii) A pure BiLSTM path (orange in Fig. 1a) dedicated to DFL identification leverages an identical single-layer BiLSTM (512 hidden units per direction) to detect evolutionarily conserved flexible linkers through long-range dependency analysis, distinct from binding IDRs [19].

#### Step 3: parallel functional prediction blocks

Final predictions are generated through six residue-level classifiers operating on the integrated feature vectors: (i) The binding-specific feature vector feeds five parallel Residual Multi-Layer Perceptron (ResMLP) blocks (Fig. 1b). Each block is specialized for a particular interaction type and incorporates skip connections to enhance training stability (see Supplementary Note 1 for architectural details). Each ResMLP block consists of two residual layers with 512 hidden units. (ii) The DFL-specific vector is processed by a dedicated, identically configured ResMLP block. Each predictor applies a sigmoid activation to convert the feature vector into position-wise propensity scores:


$$ {p}_i^k=\frac{1}{1+{e}^{-{x}_i^k}}\ \left(i=1,\dots, L;k=1,\dots, 6\right) $$

where ${x}_i^k$ denotes the ResMLP output for residue $i$ and function $k$, yielding a functional likelihood ${p}_i^k\in \left[0,1\right]$. $L$ is the sequence length. This parallelized architecture enables efficient multi-task learning while preventing feature interference across distinct functional categories. A complete specification of all architectural modules and hyperparameters is provided in Supplementary Table 2.

In summary, IDPFunNet’s architectural innovation lies in its dual-path feature decomposition strategy, which decouples the prediction of binding IDRs from DFLs while maintaining their inherent correlations. By combining global evolutionary patterns from ProtT5 with task-specific, lightweight yet effective feature extractors (CNN, BiLSTM) and predictors (ResMLP), the framework achieves superior performance in capturing IDRs’ context-dependent multifunctionality, as demonstrated in comparative analyses.

### Training process

We separately trained the hybrid CNN-BiLSTM path for binding IDR prediction and the BiLSTM-based path for DFL prediction, to optimize the IDPFunNet model. When training the hybrid CNN-BiLSTM path, we define the loss of the generic disordered binding ${Loss}_{DB}$ as below:


$$ Los{s}_{\mathrm{DB}}\left(y,\hat{y}\right)=\sum_{i=1}^5 Los{s}_i\left(y,\hat{y}\right), $$

$$ Los{s}_i\left(y,\hat{y}\right)=-\frac{1}{N}\sum_{j=1}^N\left[{y}_{ij}\log{\hat{y}}_{ij}+\left(1-{y}_{ij}\right)\log \left(1-{\hat{y}}_{ij}\right)\right]. $$

${Loss}_i\ \left(i=1,2,\cdots, 5\right)$ is the binary cross-entropy loss function, where $i$ represents a given type of disordered binding event, i.e. the IDRs interacted with protein, nucleic acid, lipid, ion, or other small molecules. We take each input sequence as a batch, and thus *N* corresponds to the total number of residues in the input sequence. $y$ represents the real labels, while $\hat{y}$ means the predicted scores. For the BiLSTM-based path, we also utilized the binary cross-entropy loss ${Loss}_6$ (with $i=6$ denoting DFLs) by following the formula for ${Loss}_i\ \left(i=1,2,\cdots, 5\right)$. We use the pytorch framework to train these two paths with an optimizer of stochastic gradient descent and a learning rate of 5e-4. We choose the network when the sum of the losses across all batches (i.e. all training sequences) is minimized.

### Evaluation metrics

IDPFunNet predicts six numeric propensity scores to quantify the likelihood of a residue located in DFLs or the IDRs binding to proteins, nucleic acids, lipids, ions, and other small molecules. Following CAID competitions [26–28], we evaluate its predictive performance using AUC, APS, and F1-max.

We first sort the predicted probabilities of the positive class to obtain a set of probability thresholds ${\left\{{p}_k\right\}}_{k=1}^m$ ($m$ is the total number of unique thresholds). Then, we calculate true positive rate (*TPR*), false positive rate (*FPR*), precision (*PRE*), and recall (*REC*) at each threshold ${p}_k$, using standard formulas:


$$ TPR=\frac{TP}{TP+ FN}= REC,\kern0.5em FPR=\frac{FP}{FP+ TN},\kern0.5em PRE=\frac{TP}{TP+ FP}. $$

*TP* (true positive) counts residues correctly classified as located in DFLs or binding IDRs; *FP* (false positive) counts incorrect positive assignments. *TN* (true negative) represents accurate negative classifications, while *FN* (false negative) denotes missed positives (e.g. binding residues misclassified as non-binding).

AUC, the area under the receiver operating characteristic (ROC) curve constructed by (*FPR*, *TPR*) points, assesses overall classification performance. A value close to 1 implies excellent performance, 0.5 indicates random guessing, and less than 0.5 shows sub-par performance.

APS refers to the weighted average of the *PRE* values at different *REC* levels, and is calculated as:


$$ \mathrm{APS}=\sum_{k=1}^m\left( RE{C}_k- RE{C}_{k-1}\ \right) PR{E}_k. $$

where $PR{E}_k$ and $RE{C}_k$ denote precision and recall at the *k*-th threshold ${p}_k$. A high APS means the model is good at finding correct instances and minimizing false detections.

F1-max means the maximum value of the F1-score across all thresholds ${p}_k\ \left(k=1,2,\dots, m\right)$. F1-score is a harmonic mean between *PRE* and *REC*, which is formulated as:


$$ \mathrm{F}1-\mathrm{score}=2\times \frac{PRE\times REC}{PRE+ REC}. $$

Generally, a higher F1-max indicates better overall classification. Additionally, F1-max often presents a strong positive correlation with APS.

IDPFunNet also offers binary predictions. Using the probability threshold $\overset{\sim }{p}$ corresponding to F1-max, residues with ${p}_i\ge \overset{\sim }{p}$ are classified as positives (disordered and functional), and others are negatives. We use MCC to assess the quality of binary predictions [19, 21, 22, 24]. MCC is calculated from all four confusion matrix elements (*TP*, *TN*, *FP*, and *FN*) as below:


$$ \mathrm{MCC}=\frac{\mathrm{TP}\times \mathrm{TN}-\mathrm{FP}\times \mathrm{FN}}{\sqrt{\left(\mathrm{TP}+\mathrm{FP}\right)\times \left(\mathrm{TP}+\mathrm{FN}\right)\times \left(\mathrm{TN}+\mathrm{FP}\right)\times \left(\mathrm{TN}+\mathrm{FN}\right)}}, $$

with values ranging from −1 to 1. A MCC of 1 means perfect prediction, 0 implies random guessing, and −1 indicates total misclassification. It is especially reliable for imbalanced datasets, and a higher value indicates better classification.

### Hypothesis testing framework

To rigorously evaluate the statistical significance of IDPFunNet’s performance advantages, we employed a hypothesis testing framework across six independent test sets using four evaluation metrics: AUC, APS, F1-max, and MCC. Our protocol used a bootstrap resampling strategy designed to handle class imbalance (the complete workflow and formulas are provided in Supplementary Note 2 and Supplementary Fig. 1). In each of the 30 iterations per test set, we constructed a subsample comprising 50% of the total sequences (K = (M + N)/2), where M and N denote the total numbers of positive and negative sequences in the dataset, respectively.

For categories with sufficient positive samples (*M* ≥ 10), sequences were randomly drawn to maintain the original class ratio within the subsample. For categories with scarce positives (*M* < 10, e.g. TE83 lipid-binding), we retained all *M* positive sequences and performed negative subsampling, randomly selecting *n* = *K* − *M* negatives to reach the target subsample size *K*. This ensured maximal use of limited positive data while controlling subset size.

This approach generated paired 30-dimensional metric vectors (IDPFunNet versus comparator) for each test set. We computed the mean and standard deviation of these vectors to quantify the overall performance and stability of each method. Prior to hypothesis testing, data normality was assessed using the Anderson–Darling test (*p*-value < 0.05). Normally distributed vectors underwent paired t-tests; otherwise, Wilcoxon signed-rank tests were used. Statistical significance was set at *p*-value < 0.05.

Notably, technical constraints limited predictions for some methods on CAID3_DFL sequences (e.g. IPA-AF2-Linker [46], ESMDisPred-2 [47]), which failed to generate predictions for 30% (6/20) of CAID3_DFL sequences and are currently inaccessible [28]. To ensure a fair comparison, hypothesis testing for CAID3_DFL was restricted to the 14 proteins with complete predictions across all methods, eliminating bias from missing data while preserving statistical power.

## Results

### Insight on the CNN-BiLSTM complementarity for IDR multifunction prediction

CNN and BiLSTM have become cornerstone architectures for protein function prediction [48–52]. The CNN architecture specializes in local motif detection [37], while BiLSTM excels at modeling long-range sequence dependencies [38]. To systematically evaluate their applicability to IDR multi-functions, we engineered standalone CNN and BiLSTM variants by substituting the original dual-path in our base model with their respective architectures (Fig. 1). Performance was quantified via AUC and APS metrics on the TE210 benchmark.

**Figure 2:**
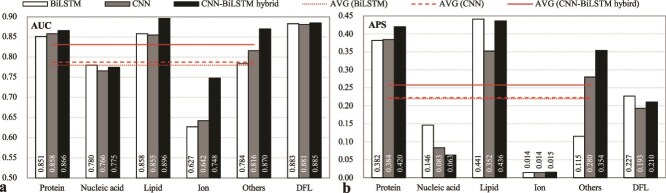
The predictive quality of the CNN-based, BiLSTM-based and the CNN-BiLSTM hybrid models, which is evaluated by using (a) AUC and (b) APS metrics on the test set TE210. These models were developed by replacing the original dual-path architecture in our base model IDPFunNet with their respective architectures. The “others” category represents IDRs binding to other small molecules. The cross-category average AVG (*) aggregates performance across five binding classes: Protein-, nucleic acid-, lipid-, ion-, and other small molecule-binding IDRs.

Comparative analysis (Fig. 2) revealed both architectures achieved reasonable predictive performance (DFL prediction: AUC ~0.88, APS 0.193–0.227; binding IDRs: AUC ~0.78, APS ~0.22). We observed a task-dependent performance profile: BiLSTM showed relative advantages for nucleic acid and lipid binding (with AUC gains of 0.4%–1.8% and APS gains of 25.3%–75.9% over the alternative), whereas CNN proved particularly effective for protein and other small-molecule detection (with corresponding gains of 0.8%–4.1% in AUC and 0.5%–143.5% in APS). This specialization aligns with their inherent computational strengths—local pattern recognition versus global context integration.

Hybridization of these architectures yielded significant improvements: The CNN-BiLSTM ensemble surpassed standalone models by 0.93%–19.3% AUC across most binding tasks, with average AUC and APS improvements of ≥5.5% and ≥15.7%, respectively (Fig. 2). Interestingly, for DFL prediction, BiLSTM alone matched the CNN-BiLSTM hybrid’s AUC performance (~0.88) while achieving ≥8.1% higher APS. These findings highlight CNN’s complementary role to BiLSTM in binding IDR prediction and underscore its limited contribution to the DFL task.

Guided by the structural dichotomy between interaction-prone and linker IDRs [27], we formalized this specialization through IDPFunNet’s dual-path architecture: CNN-BiLSTM hybrids for binding IDR prediction and dedicated BiLSTM streams for DFL annotation.

### ProtT5 outperforms ESM-family models and AlphaFold2 in feature encoding

Recent advances in PLMs have revolutionized IDP research by capturing evolutionary and biophysical signatures at residue-level resolution [35, 36, 53]. While PLMs like ESM-1b/2 and ProtT5 excel in sequence-based functional motif prediction [32–36], AF2 provides structural insights through atomic-level modeling [27, 42, 54]. This dual perspective—sequence-derived embeddings versus structure-based features—motivated our systematic comparison to identify optimal representations for multi-category IDR function annotation. Details of PLMs and AF2-derived features are provided in Supplementary Notes 3 and 4, respectively.

**Figure 3:**
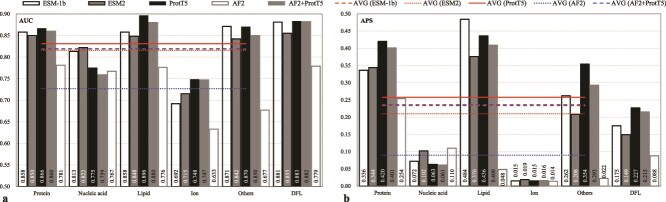
Performance comparison of five IDPFunNet variants on the TE210 benchmark. (a) AUC and (b) APS metrics demonstrate differential predictive capabilities across six functional categories of IDRs. The “others” category represents IDRs binding to other small molecules. The cross-category average AVG (*) aggregates performance across five binding classes: protein-, nucleic acid-, lipid-, ion-, and other small molecule-binding IDRs. Model variants employ distinct sequence encoding strategies: three implementations using pretrained language model embeddings (ESM-1b/ESM2/ProtT5) versus two structure-based approaches using AF2-derived features either independently (i.e. AF2) or concatenated with ProtT5 embeddings (i.e. AF2 + ProtT5).

Benchmarking three PLM-integrated frameworks (IDPFunNet-ESM-1b, -ESM2, -ProtT5) on TE210 revealed architecture-dependent specialization (Fig. 3a and b). ProtT5 demonstrated cross-category superiority, achieving ≥0.9% higher AUC for protein-binding and ≥4.4% AUC improvement for lipid-binding IDRs, with DFL prediction APS exceeding ESM models by 29.7%. Conversely, ESM2 excelled in the nucleic acid-binding task (≥1.1% AUC gain). ProtT5’s average performance surpassed ESM-family models by ≥1.5% AUC and 10.2% APS across binding IDR categories, establishing its broad applicability.

Comparative analysis with AF2-derived features highlighted ProtT5’s dominance in five of six functional categories (Fig. 3a and b). ProtT5 achieved significantly higher AUC in protein-binding (0.866 versus 0.781), lipid-binding (0.896 versus 0.776), and DFL prediction (0.883 versus 0.779), with APS values greater in other small molecule-binding (0.354 versus 0.022). Although AF2 showed marginal advantages in nucleic acid-binding (AUC: 0.775 versus 0.767) and ion-binding (APS: 0.015 versus 0.014), the ProtT5 + AF2 hybrid model consistently underperforms ProtT5 alone by a relatively small margin (relative differences ≤2.3% in AUC), suggesting limited complementarity between sequence and structural embeddings.

In summary, ProtT5 demonstrates robust cross-category performance (average AUC improvement: 1.2%–14.2%), with its encoder-decoder architecture proving particularly effective in capturing conformational plasticity essential for IDR multifunctional annotation. This enhanced capacity to represent structurally dynamic regions establishes ProtT5 as the optimal choice for IDPFunNet’s sequence encoding framework, where evolutionary-biophysical feature integration drives prediction accuracy.

### Multi-task learning enhances prediction robustness for binding IDR prediction

Existing approaches for IDR function prediction predominantly employ single-task architectures optimized for individual binding types [25]. This specialization limits their capacity to exploit shared functional patterns across interaction categories.

**Figure 4:**
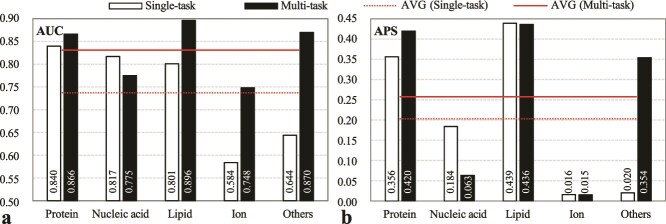
Performance of single-task and multi-task models across five binding types of IDRs, which is evaluated by using (a) AUC and (b) APS metrics on the test set TE210. The “others” category represents IDRs binding to other small molecules. AVG (*) aggregates performance across five binding classes: protein-, nucleic acid-, lipid-, ion-, and other small molecule-binding IDRs.

Our comparison of single- versus multi-task architectures across five functional categories (protein/nucleic acid/lipid/ion/other small molecule-binding) reveals critical performance tradeoffs; please refer to Fig. 4. The multi-task model demonstrated superior AUC in four categories: protein-binding (3.1% improvement), lipid-binding (11.9% increase), ion-binding (28.1% gain), and other small molecule-binding (35.1% higher). Conversely, the single-task architecture excelled in nucleic acid-binding (AUC: 0.817 versus 0.775). APS analysis revealed multi-task dominance in protein-binding (0.42 versus 0.356) and other small molecule-binding (0.354 versus 0.020), while the single-task model achieved higher APS in nucleic acid-binding (0.184 versus 0.063).

Aggregated across all categories, multi-task learning achieved a 12.7% higher mean AUC and 26.9% greater mean APS compared to the single-task framework. These improvements reflect enhanced feature sharing across correlated tasks, particularly evident in other small molecule-binding where cross-task correlations are strongest.

Therefore, IDPFunNet adopts the multi-task paradigm to balance comprehensive functional coverage with biological fidelity.

### Performance of attention mechanism for IDR multifunction prediction

**Figure 5:**
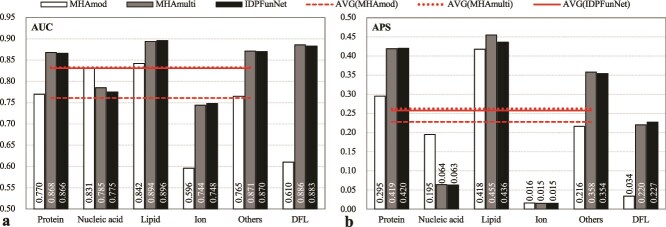
Performance of three models MHAmod, MHAmulti and IDPFunNet across six functional categories of IDRs, which is evaluated by using (a) AUC and (b) APS metrics on the test set TE210. The ‘others’ category represents IDRs binding to other small molecules. AVG (*) aggregates performance across five binding classes: protein-, nucleic acid-, lipid-, ion-, and other small molecule-binding IDRs.

The multi-head attention (MHA) mechanism uniquely captures long-range sequential dependencies while maintaining local patterns through positional encoding [43]. Leveraging its demonstrated success in protein structure prediction via transformer frameworks [54], we evaluated MHA’s capacity for IDR functional analysis through two engineered variants: (i) MHAmod replaces IDPFunNet’s dual-path architecture with pure MHA modules, and (ii) MHAmulti substitutes the dual-path system with a hybrid of multi-scale CNNs (kernel sizes: 1, 3, 5, 7) and MHA—a design proven to extract hierarchical features [24]. These comparative models were benchmarked against the original IDPFunNet across six functional categories using the TE210 test set, with performance quantified by AUC and APS metrics (Fig. 5).

Comparative evaluation, shown in Fig. 5, revealed distinct performance patterns across functional categories. For binding IDRs, IDPFunNet, and MHAmulti achieved higher mean performance than MHAmod, with average AUC and APS improvements of ≥9.2% and ≥13%, respectively. This advantage also extended to DFL prediction, with IDPFunNet and MHAmulti achieving higher AUC (0.883 and 0.886 versus 0.610) and APS (0.227 and 0.22 versus 0.034) than MHAmod. Detailed category-specific analysis showed consistent dominance in protein- (AUC: 0.87 versus 0.77; APS: 0.42 versus 0.30), lipid- (AUC: 0.89 versus 0.84; APS: 0.436–0.455 versus 0.418), and other small molecule-binding IDRs (AUC: 0.87 versus 0.77; APS: 0.35 versus 0.22). Notably, MHAmod exhibited exclusive superiority in nucleic acid-binding IDRs (AUC: 0.831 versus 0.78; APS: 0.195 versus 0.06), while ion-binding predictions remained challenging for all models (APS < 0.02). Despite near-equivalent performance between IDPFunNet and MHAmulti (relative differences ≤1.3% in AUC and ≤4.2% in APS), the former’s streamlined architecture with reduced parameter complexity offers practical implementation benefits.

In brief, our systematic comparison identifies IDPFunNet as the optimal framework balancing predictive power and architectural efficiency.

### Benchmarking against SOTA methods across IDR functional categories

We evaluated IDPFunNet’s predictive accuracy for DFLs and five binding IDR types across two independent test sets (TE210/TE83), benchmarking against three SOTA predictors: DisoFLAG [36], DeepDISOBind [24], and DisoLipPred [25]. DisoFLAG was stated to outperform leading methods in CAID2 [36], and was reported as the top-ranked method in the latest CAID3 (https://caid.idpcentral.org/challenge/results). It employs a PLM and graph-based interactive architecture to systematically predict DFLs and IDRs interacting with proteins, DNA, RNA, ions, and lipids. DeepDISOBind, a deep convolutional network, concurrently predicts IDRs binding to RNA, DNA, and proteins. DisoLipPred, a bidirectional recurrent neural network, specializes in lipid-binding IDR prediction. Since DisoFLAG and DeepDISOBind are designed to predict DNA-binding and RNA-binding IDRs separately, we combined their prediction scores and binary classifications as the results for nucleic acid binding for a unified evaluation. Please refer to Supplementary Note 5 for details.

**Figure 6:**
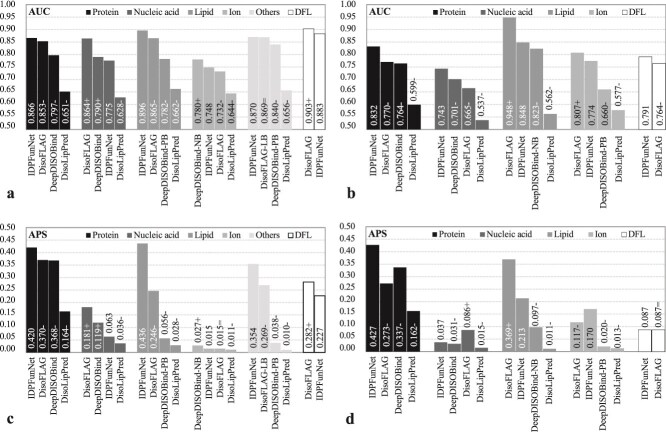
Comparative performance of IDPFunNet and SOTA methods on independent test sets TE210 (a, c) and TE83 (b, d), as measured by AUC (a, b) and APS (c, d). For lipid-, ion-, and other small molecule-binding IDR predictions, we evaluated model variants DeepDISOBind-PB, DeepDISOBind-NB, and DisoFLAG-LB, where PB/NB/LB denote protein/nucleic acid/lipid binding. These variants correspond to task-specific components of DeepDISOBind and DisoFLAG frameworks. Notably, other small molecule-binding IDR annotations are unavailable in TE83. “+”/“−” next to a given AUC and APS value indicates that the corresponding method is significantly better/worse than IDPFunNet (*p*-value < 0.05).

IDPFunNet demonstrates robust performance across IDR functional categories, as evidenced by the computational analysis from the TE210 and TE83 datasets (Fig. 6, and Supplementary Figs 2 and 3). The detailed performance metrics, including their means and standard deviations across bootstrap iterations, are provided in Supplementary Table 3. A synthesized summary highlighting statistical significance against comparator methods is presented in Supplementary Table 4. On TE210, IDPFunNet attained AUC, APS, and F1-max ranges of 0.748–0.896, 0.015–0.436, and 0.035–0.477, respectively. On TE83, these metrics range from 0.743 to 0.848 (AUC), 0.037 to 0.427 (APS), and 0.107 to 0.450 (F1-max). Holistic evaluation across AUC, APS, F1-max, and MCC revealed that IDPFunNet achieved the highest accuracy for protein binding (AUC ≥0.832, APS ≥0.42, F1-max ≥0.45, MCC ≥0.355), followed by lipid binding (AUC ≥ 0.848, APS ≥0.213, F1-max ≥0.351, MCC ≥0.217), while performance was suboptimal for nucleic acid and ion binding (AUC ≤0.774, APS ≤0.170, F1-max ≤0.276, MCC ≤0.248).

Comparative analysis with SOTA methods revealed IDPFunNet’s task-specific superiority, achieving dominant performance in protein-binding prediction while maintaining competitive accuracy for other binding types and DFLs. As detailed in Fig. 6, and Supplementary Figs 2 and 3 and Supplementary  Table  4, IDPFunNet attained peak metrics for protein-binding IDRs across both TE210 and TE83 datasets, outperforming SOTA methods by significant margins (improvements: $\ge$1.5% AUC, $\ge$13.5% APS, $\ge$4.8% F1-max, $\ge$7.5% MCC). This advantage extended to lipid-binding prediction on TE210, where IDPFunNet achieved 0.896 AUC/0.436 APS/0.454 F1-max/0.43 MCC - surpassing DisoFLAG, DeepDISOBind-PB, and DisoLipPred by ≥3.6% AUC, ≥77.2% APS, ≥48.9% F1-max, and 175.6% MCC. Nucleic acid-binding performance diverged, with IDPFunNet (AUC: 0.775) trailing DisoFLAG (AUC: 0.864) and DeepDISOBind (AUC: 0.79) on TE210 but showing at least 6% AUC gain on TE83. Notably, IDPFunNet achieved cross-dataset enhancements on TE83: 6% AUC gain for nucleic acid-binding, 43.5% APS improvement for ion-binding, and 3.5% AUC increase for DFLs, alongside 0.149–2.179 times MCC boosts across five binding classes (*p*-value < 0.05). This advantage is statistically robust, with performance stability across resampling iterations documented in Supplementary Table 3.

Moreover, IDPFunNet shows good robustness in our multi-class classification tasks across the TE210 and TE83 datasets (the latter TE83 being a recently updated functional IDR dataset). As illustrated in Fig. 6 and Supplementary Table 4, IDPFunNet and DeepDISOBind exhibited superior stability in protein binding prediction, with all four metrics’ variations ≤0.05 across datasets. For nucleic acid binding, IDPFunNet showed smaller performance fluctuations (AUC variation <0.05) compared to DeepDISOBind and DisoFLAG, the latter displaying the largest AUC variation (~0.2). In lipid binding, stability varied by metric: IDPFunNet maintained AUC stability (variation <0.05) but showed APS/F1-max shifts >0.1, while DisoFLAG had minimal MCC variation (0.016) but larger AUC/APS/F1-max changes (0.083–0.138). For DFL prediction, both IDPFunNet and DisoFLAG exhibited significant metric fluctuations (>0.05 variation in all metrics). Collectively, IDPFunNet demonstrated enhanced stability for protein and nucleic acid binding compared to SOTA alternatives.

To ensure a fair comparison independent of training data scale, we retrained DisoFLAG and DeepDISOBind on our datasets (DisoFLAG* and DeepDISOBind*; Supplementary Note 6). Their evaluated performance (Supplementary Tables 3 and 4, Supplementary Fig. 6) showed only limited and inconsistent gains (e.g. improved in protein-binding but declined in DFL prediction). This confirms that IDPFunNet’s superiority stems primarily from its architecture, not merely larger training data.

IDPFunNet establishes superior performance in IDR multifunction prediction, particularly excelling in protein/lipid interaction modeling. Although nucleic acid-binding prediction remains challenging, its cross-task generalizability and dataset adaptability outperform alternatives. The framework demonstrates enhanced robustness for core interaction types despite inherent limitations in data-scarce categories (ion-binding) and class-imbalanced scenarios. These findings position IDPFunNet as a versatile solution for IDR multifunctional annotation, balancing specialized accuracy with broad applicability.

### Benchmarking against CAID top performers for binding IDR and DFL prediction

**Figure 7:**
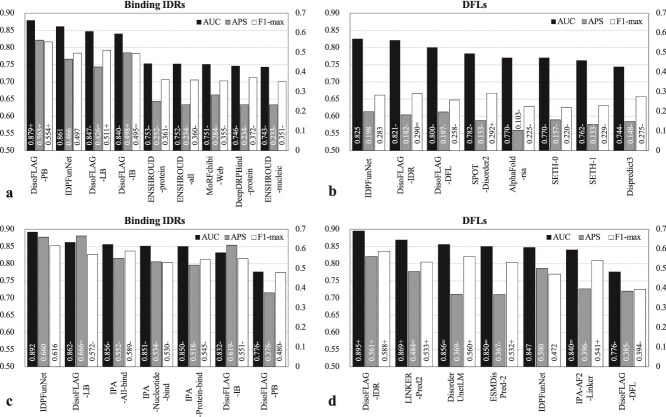
Performance evaluation of IDPFunNet on CAID2 (a, b) and CAID3 (c, d) blind test datasets. The *x*-axes present the top five AUC-ranked methods in CAID competitions for binding IDR (a, c) and DFL (b, d) prediction, alongside DisoFLAG’s top 3 components (a, c). AUC values are displayed on the primary *y*-axes; APS and F-max on the secondary *y*-axes. The model variants DisoFLAG-LB/IB/PB/DFL denote task-specific components of the DisoFLAG framework, where PB/LB/IB is the abbreviation of protein/lipid/ion binding. “+”/“−” next to a metric value indicates that the corresponding method is significantly better/worse than IDPFunNet (*p*-value < 0.05). Notably, (d) summarizes the results for 14 sequences extracted from CAID3_DFL, i.e. denoted as CAID3_DFL14 set, as all methods listed on the *x*-axis can generate DFL predictions for them.

To rigorously evaluate IDPFunNet’s predictive capacity for binding IDRs and DFLs, we established a benchmarking framework using four CAID blind test sets (CAID2/3_Bind and CAID2/3_DFL), comparing against the top five AUC-ranked methods from CAID2/3. Prediction results for reference methods were retrieved from the CAID portal (https://caid.idpcentral.org/challenge/results). CAID3 prediction results are based on the January 2025 release. For binding IDR assessment, we exclusively utilized IDPFunNet’s protein-binding module due to its demonstrated stability across TE210/TE83 benchmarks. Similarly, only the DFL-specific component was evaluated on CAID2/3_DFL sets. Given DisoFLAG’s inconsistent validation performance, we assessed all its binding IDR modules on CAID2/3_bind but reported only the top three performers (by AUC) in Fig. 7 and Supplementary Table 5. For DFL comparisons, we included DisoFLAG’s top-ranked DFL predictor DisoFLAG_IDR in CAID3 alongside its dedicated DFL module (DisoFLAG_DFL) to ensure equitable evaluation on CAID2/3_DFL (Fig. 7, Supplementary Figs 4 and 5 and Supplementary  Table  5).

On CAID2 benchmarks (Fig. 7a-b, Supplementary Fig. 4 and Table 5), IDPFunNet achieved significant performance gains over all top-five methods (binding: ENSHROUD-protein/all/nucleic, MoRFchibi-Web, DeepDRPBind-protein; DFL: SPOT-Disorder2, AlphaFold-rsa, SETH-0/1, Dispredict3), with minimum relative improvements of 14.3% in AUC and 64.1% in APS. Compared to DisoFLAG, IDPFunNet matched the best-performing DisoFLAG-PB component in binding AUC while surpassing both DisoFLAG-IDR and DisoFLAG-DFL in DFL metrics (0.5% higher AUC and APS).

For CAID3_Bind (Fig. 7c, Supplementary Fig. 5 and Table  5), IDPFunNet outperformed the top five CAID3 methods (DisoFLAG-LB/PB, IPA-All/Nucleotide/Protein-bind) and DisoFLAG’s leading components across almost all metrics, achieving 3.5%–14.9% higher AUC and 4.6%–28.3% higher F1-max, while APS changes ranged from −0.9% to 75.5%. In CAID3_DFL assessment (Supplementary Table 5), IDPFunNet exhibited 2.1%/16.7%/22.2% improvements in AUC/APS/F1-max over DisoFLAG-DFL, though its AUC trailed top CAID3 methods (IPA-AF2-Linker, DisoFLAG-IDR, etc.) by 1.3%–4.8% while exceeding their APS by 13.3%–65.5%. Notably, critical limitations emerged: leading DFL predictors (IPA-AF2-Linker, ESMDisPred-2) failed to process long sequences (Supplementary Table 5), prompting evaluation on the CAID3_DFL14 subset (14/20 fully predicted proteins). This adjustment altered method rankings (e.g., IPA-AF2-Linker AUC decreased from 0.881 to 0.84), with all reported differences remaining statistically significant (*p*-value < 0.05; Fig. 7d, Supplementary Fig. 5 and Table  5). 

Collectively, IDPFunNet demonstrated robust cross-platform consistency, maintaining consistently high rankings for binding IDR prediction while delivering competitive DFL accuracy (CAID2/3). Intriguingly, generic IDR predictors (DisoFLAG-IDR, SETH series) consistently outperformed DFL-specialized tools (DisoFLAG-DFL, IPA-AF2-Linker), potentially reflecting incomplete DFL annotations in current databases [55].

## Conclusion

IDPFunNet introduces a hybrid deep learning framework for systematic prediction of six IDR functions, encompassing DFLs and five binding subtypes (protein/nucleic acid/lipid/ion/other small molecule). The architecture employs a dual-path design: (i) a CNN-BiLSTM hybrid capturing local motifs and long-range dependencies for multi-class binding predictions, and (ii) a dedicated BiLSTM network optimized for DFL annotation. This structural specialization addresses the heterogeneous nature of IDR functions through parallel processing of distinct feature hierarchies.

Benchmarking across six datasets (including CAID2/3 blind tests) demonstrates IDPFunNet’s superior performance in protein IDR prediction, achieving AUCs >0.83 with 13.5%–26.7% APS improvements over SOTA methods (*p*-value < 0.05). The model consistently ranked top-two in CAID competitions, validating cross-dataset robustness. For DFL annotation, IDPFunNet maintained competitive accuracy (AUC: 0.791–0.883) across datasets, validating its generalizability.

A key innovation involves ProtT5-based feature engineering, where evolutionary-biophysical residue embeddings outperformed ESM-family models and AF2 structural features. Integration of ProtT5 enhanced prediction stability (cross-dataset AUC variation ≤0.05) while boosting average AUC/APS by 1.5%/10.2%, respectively. This establishes PLMs as critical for encoding context-dependent binding signatures.

Current limitations include moderate nucleic acid-binding prediction (AUC: 0.743–0.775), where single-task architectures with ESM2 embeddings show superior performance, suggesting subtype-specific optimization needs. Class imbalance in lipid/ion-binding datasets (e.g. TE83: <10 positives) further constrained APS reliability, necessitating synthetic minority oversampling techniques. Future iterations may integrate attention mechanisms for nucleic acid interactions and active learning strategies to address data scarcity.

As a novel framework concurrently addressing multiple IDR functions, IDPFunNet advances disorder biology research through three core strengths: (i) hybrid architecture balancing local/global feature extraction, (ii) language model-enhanced evolutionary profiling, and (iii) modular design adaptable to emerging functional categories. These innovations position it as a versatile tool for decoding protein disorder-function relationships, with potential applications in pathogenic mutation interpretation and targeted drug discovery.

**Key Points**

- IDPFunNet introduces a dual-path neural design that concurrently processes local binding motifs (via CNNs) and long-range dependencies (via BiLSTMs), while employing a dedicated BiLSTM branch for DFL prediction, to effectively address functional heterogeneity.
- ProtT5 embeddings enhanced performance, surpassing ESM-family models and AF2-derived structural features, and demonstrating residue-level evolutionary-structural embeddings’ critical role in capturing context-dependent functional signatures of IDRs.
- IDPFunNet demonstrates enhanced robustness compared to state-of-the-art alternatives across six independent test sets, with top rankings in CAID2/3 challenges.

## Supplementary Material

Supplement_revision_submitted_bbag126

## Data Availability

The source code of IDPFunNet is freely available as a standalone software package at: https://github.com/IDRIDP/IDPFunNet/tree/master. A user-friendly webserver offering free access to IDPFunNet is available at: https://yanglab.qd.sdu.edu.cn/IDPFunNet/. This platform allows users to submit protein sequences and obtain predictions without local installation. All benchmark datasets used in this study (TR552, VA227, TE210, TE83, CAID2, and CAID3) are publicly accessible and can be downloaded from: https://yanglab.qd.sdu.edu.cn/IDPFunNet/benchmark/.

## References

[1] Oldfield CJ, Dunker AK. Intrinsically disordered proteins and intrinsically disordered protein regions. *Annu Rev Biochem* 2014;83:553–84. https://doi.org/annurev-biochem-072711-164947 24606139 10.1146/annurev-biochem-072711-164947

[2] Uversky VN . Introduction to intrinsically disordered proteins (IDPs). *Chem Rev* 2014;114:6557–60. 10.1021/cr500288y 25004990

[3] van der Lee R, Buljan M, Lang B et al. Classification of intrinsically disordered regions and proteins. *Chem Rev* 2014;114:6589–631. 10.1021/cr400525m 24773235 PMC4095912

[4] Wright PE, Dyson HJ. Intrinsically disordered proteins in cellular signalling and regulation. *Nat Rev Mol Cell Biol* 2015;16:18–29. 10.1038/nrm3920 25531225 PMC4405151

[5] Babu MM . The contribution of intrinsically disordered regions to protein function, cellular complexity, and human disease. *Biochem Soc Trans* 2016;44:1185–200. 10.1042/BST20160172 27911701 PMC5095923

[6] Holehouse AS, Kragelund BB. The molecular basis for cellular function of intrinsically disordered protein regions. *Nat Rev Mol Cell Biol* 2024;25:187–211. 10.1038/s41580-023-00673-0 37957331 PMC11459374

[7] Fung HYJ, Birol M, Rhoades E. IDPs in macromolecular complexes: the roles of multivalent interactions in diverse assemblies. *Curr Opin Struct Biol* 2018;49:36–43. 10.1016/j.sbi.2017.12.007 29306779 PMC5915967

[8] Orand T, Jensen MR. Binding mechanisms of intrinsically disordered proteins: insights from experimental studies and structural predictions. *Curr Opin Struct Biol* 2025;90:102958. 10.1016/j.sbi.2024.102958 39740355

[9] Uversky VN . Intrinsic disorder, protein-protein interactions, and disease. *Adv Protein Chem Struct Biol* 2018;110:85–121. 10.1016/bs.apcsb.2017.06.005 29413001

[10] Raj N, Attardi LD. The transactivation domains of the p53 protein. *Cold Spring Harb Perspect Med* 2017;7:a026047. 10.1101/cshperspect.a026047 PMC5204331 27864306

[11] Hilser VJ, Thompson EB. Intrinsic disorder as a mechanism to optimize allosteric coupling in proteins. *Proc Natl Acad Sci U S A* 2007;104:8311–5. 10.1073/pnas.0700329104 17494761 PMC1895946

[12] Ferreon ACM, Ferreon JC, Wright PE et al. Modulation of allostery by protein intrinsic disorder. *Nature* 2013;498:390–4. 10.1038/nature12294 23783631 PMC3718496

[13] Terakawa T, Higo J, Takada S. Multi-scale ensemble Modeling of modular proteins with intrinsically disordered linker regions: application to p53. *Biophys J* 2014;107:721–9. 10.1016/j.bpj.2014.06.026 25099811 PMC4129485

[14] Wang YF, Fisher JC, Mathew R et al. Intrinsic disorder mediates the diverse regulatory functions of the Cdk inhibitor p21. *Nat Chem Biol* 2011;7:214–21. 10.1038/nchembio.536 21358637 PMC3124363

[15] Mészáros B, Simon I, Dosztányi Z. Prediction of protein binding regions in disordered proteins. *PLoS Comput Biol* 2009;5:5. 10.1371/journal.pcbi.1000376 PMC2671142 19412530

[16] Peng ZL, Kurgan L. High-throughput prediction of RNA, DNA and protein binding regions mediated by intrinsic disorder. *Nucleic Acids Res* 2015;43:e121. 10.1093/nar/gkv585 26109352 PMC4605291

[17] Malhis N, Jacobson M, Gsponer J. MoRFchibi SYSTEM: software tools for the identification of MoRFs in protein sequences. *Nucleic Acids Res* 2016;44:W488–93. 10.1093/nar/gkw409 27174932 PMC4987941

[18] Meng F, Kurgan L. DFLpred: high-throughput prediction of disordered flexible linker regions in protein sequences. *Bioinformatics* 2016;32:i341–50. 10.1093/bioinformatics/btw280 27307636 PMC4908364

[19] Peng Z, Xing Q, Kurgan L. APOD: accurate sequence-based predictor of disordered flexible linkers. *Bioinformatics* 2020;36:i754–61. 33381830 10.1093/bioinformatics/btaa808 PMC7773485

[20] Perovic V, Sumonja N, Marsh LA et al. IDPpi: protein-protein interaction analyses of human intrinsically disordered proteins. *Sci Rep* 2018;8:8. 10.1038/s41598-018-28815-x 30002402 PMC6043496

[21] Peng Z, Li Z, Meng Q et al. CLIP: accurate prediction of disordered linear interacting peptides from protein sequences using co-evolutionary information. *Brief Bioinform* 2023;24:bbac502. 10.1093/bib/bbac502 36458437

[22] Hu G, Katuwawala A, Wang K et al. flDPnn: accurate intrinsic disorder prediction with putative propensities of disorder functions. *Nat Commun* 2021;12:4438. 10.1038/s41467-021-24773-7 34290238 PMC8295265

[23] Wang K, Hu G, Basu S et al. flDPnn2: accurate and fast predictor of intrinsic disorder in proteins. *J Mol Biol* 2024;436:168605. 10.1016/j.jmb.2024.168605 39237195

[24] Zhang F, Zhao B, Shi W et al. DeepDISOBind: accurate prediction of RNA-, DNA- and protein-binding intrinsically disordered residues with deep multi-task learning. *Brief Bioinform* 2022;23:23. 10.1093/bib/bbab521 34905768

[25] Katuwawala A, Zhao B, Kurgan L. DisoLipPred: accurate prediction of disordered lipid-binding residues in protein sequences with deep recurrent networks and transfer learning. *Bioinformatics* 2021;38:115–24. 10.1093/bioinformatics/btab640 34487138

[26] Necci M, Piovesan D, Hoque MT et al. Critical assessment of protein intrinsic disorder prediction. *Nat Methods* 2021;18:472–81. 10.1038/s41592-021-01117-3 33875885 PMC8105172

[27] Del Conte A, Mehdiabadi M, Bouhraoua A et al. Critical assessment of protein intrinsic disorder prediction (CAID) - results of round 2. *Proteins Struct Funct Bioinform* 2023;91:1925–34. 10.1002/prot.26582 37621223

[28] Mehdiabadi M, Del Conte A, Nugnes MV et al. Critical assessment of protein intrinsic disorder round 3-predicting disorder in the era of protein language models. *Proteins* 2026;94:414–24. 10.1002/prot.70045 40859602 PMC12750029

[29] Sharma R, Tsunoda T, Sharma A. DRPBind: prediction of DNA, RNA and protein binding residues in intrinsically disordered protein sequences, bioRxiv. 2023. 2023.2003.2020.533427.

[30] Piovesan D, Monzon AM, Tosatto SCE. Intrinsic protein disorder and conditional folding in AlphaFoldDB. *Protein Sci* 2022;31:e4466. 10.1002/pro.4466 36210722 PMC9601767

[31] Hanson J, Paliwal KK, Litfin T et al. SPOT-Disorder2: improved protein intrinsic disorder prediction by Ensembled deep learning. *Genomics Proteomics Bioinformatics* 2019;17:645–56. 10.1016/j.gpb.2019.01.004 32173600 PMC7212484

[32] Rives A, Meier J, Sercu T et al. Biological structure and function emerge from scaling unsupervised learning to 250 million protein sequences. *Proc Natl Acad Sci U S A* 2021;118:e2016239118. 10.1073/pnas.2016239118 PMC8053943 33876751

[33] Lin Z, Akin H, Rao R et al. Evolutionary-scale prediction of atomic-level protein structure with a language model. *Science* 2023;379:1123–30. 10.1126/science.ade2574 36927031

[34] Elnaggar A, Heinzinger M, Dallago C et al. ProtTrans: toward understanding the language of life through self-supervised learning. *IEEE Trans Pattern Anal Mach Intell* 2022;44:7112–27. 10.1109/TPAMI.2021.3095381 34232869

[35] Xu S, Onoda A. Accurate and fast prediction of intrinsically disordered protein by multiple protein language models and ensemble learning. *J Chem Inform Model* 2023;64:2901–11. 10.1021/acs.jcim.3c01202 37883249

[36] Pang Y, Liu B. DisoFLAG: accurate prediction of protein intrinsic disorder and its functions using graph-based interaction protein language model. *BMC Biol* 2024;22:3. 10.1186/s12915-023-01803-y 38166858 PMC10762911

[37] Krizhevsky A, Sutskever I, Hinton GE. ImageNet classification with deep convolutional neural networks. *Commun Acm* 2017;60:84–90. 10.1145/3065386

[38] Huang Z, Xu W, Yu K. *Bidirectional LSTM-CRF models for sequence tagging*, arXiv e-prints, 2015. arXiv:1508.01991.

[39] Chakrabarti P, Chakravarty D. Intrinsically disordered proteins/regions and insight into their biomolecular interactions. *Biophys Chem* 2022;283:106769. 10.1016/j.bpc.2022.106769 35139468

[40] Dunker AK, Brown CJ, Lawson JD et al. Intrinsic disorder and protein function. *Biochemistry* 2002;41:6573–82. 10.1021/bi012159+ 12022860

[41] Fatafta H, Samantray S, Sayyed-Ahmad A et al. Molecular simulations of IDPs: from ensemble generation to IDP interactions leading to disorder-to-order transitions. *Prog Mol Biol Trans Sci* 2021;183:135–85. 10.1016/bs.pmbts.2021.06.003 34656328

[42] Guo HB, Perminov A, Bekele S et al. AlphaFold2 models indicate that protein sequence determines both structure and dynamics. *Sci Rep* 2022;12:10696. 10.1038/s41598-022-14382-9 35739160 PMC9226352

[43] Vaswani A, Shazeer N, Parmar N et al. Attention is all you need. In: Guyon I, Luxburg UV, Bengio S et al. (eds). Advances in Neural Information Processing Systems 30 (NIPS 2017), Vol. 30. La Jolla, CA: Neural Information Processing Systems (NIPS), 2017.

[44] Aspromonte MC, Nugnes MV, Quaglia F et al. DisProt in 2024: improving function annotation of intrinsically disordered proteins. *Nucleic Acids Res* 2024;52:D434–41. 10.1093/nar/gkad928 37904585 PMC10767923

[45] Li WZ, Godzik A. Cd-hit: a fast program for clustering and comparing large sets of protein or nucleotide sequences. *Bioinformatics* 2006;22:1658–9. 10.1093/bioinformatics/btl158 16731699

[46] Malhis N . Probabilistic annotations of protein sequences for intrinsically disordered features, bioRxiv. 2025. 2024.2012.2018.629275.

[47] Kabir MWU, Dey A, Nafees F et al. ESMDisPred: a structure-aware CNN-transformer architecture for intrinsically disordered protein prediction, bioRxiv. 2026. 2026.2001.2022.701204.

[48] Kulmanov M, Hoehndorf R. DeepGOPlus: improved protein function prediction from sequence. *Bioinformatics* 2020;36:422–9. 10.1093/bioinformatics/btz595 31350877 PMC9883727

[49] Dhanuka R, Singh JP, Tripathi A. A comprehensive survey of deep learning techniques in protein function prediction. *IEEE/ACM Trans Comput Biol Bioinform* 2023;20:2291–301. 10.1109/TCBB.2023.3247634 37027658

[50] Zhu YH, Zhang CX, Yu DJ et al. Integrating unsupervised language model with triplet neural networks for protein gene ontology prediction. *PLoS Comput Biol* 2022;18:18. 10.1371/journal.pcbi.1010793 PMC9822105 36548439

[51] Ke J, Zhao J, Li H et al. Prediction of protein N-terminal acetylation modification sites based on CNN-BiLSTM-attention model. *Comput Biol Med* 2024;174:108330. 10.1016/j.compbiomed.2024.108330 38588617

[52] Fan Z, Xu Y. Predicting the functional changes in protein mutations through the application of BiLSTM and the self-attention mechanism. *Ann Data Sci* 2024;11:1077–94. 10.1007/s40745-024-00530-7

[53] Ilzhofer D, Heinzinger M, Rost B. SETH predicts nuances of residue disorder from protein embeddings. *Front Bioinform* 2022;2:1019597. 10.3389/fbinf.2022.1019597 36304335 PMC9580958

[54] Jumper J, Evans R, Pritzel A et al. Highly accurate protein structure prediction with AlphaFold. *Nature* 2021;596:583–9. 10.1038/s41586-021-03819-2 34265844 PMC8371605

[55] Wang K, Hu G, Wu ZH et al. Assessment of disordered linker predictions in the CAID2 experiment. *Biomolecules* 2024;15:14. 10.3390/biom15010014 38540707 PMC10968229

