# Supplementary material for: Hybrid deep learning with protein language models and dual-path architecture for predicting IDP functions
Source: Brief Bioinform. 2026 Apr 1;27(2):bbag126. doi: 10.1093/bib/bbag126 (PMC13043015; doi:10.1093/bib/bbag126)
Supplement: Supplement_revision_submitted_bbag126 [file supplement_revision_submitted_bbag126.docx]

**Supplement for “Hybrid Deep Learning with Protein Language Models and Dual-Path Architecture for Predicting IDP Functions”**

Jiahui Liang^1^, Yuxian Luo^1^, Baoquan Su^1^ and Zhenling Peng^1*^

^1^MOE Frontiers Science Center for Nonlinear Expectations, Research Center for Mathematics and Interdisciplinary Sciences, Shandong University, Qingdao 266237, China

^*^Corresponding authors: Zhenling Peng at [zhenling@email.sdu.edu.cn](mailto:zhenling@email.sdu.edu.cn)

# Supplementary Note 1: Introduction and configuration of CNN, BiLSTM and ResMLP in IDPFunNet

Convolutional Neural Networks (CNNs), Bidirectional Long Short-Term Memory networks (BiLSTMs), and Residual Multi-Layer Perceptrons (ResMLPs) are cornerstone deep learning architectures in bioinformatics, chosen for IDPFunNet due to their complementary strengths in modeling protein sequence data [1-3]. Their specific, relatively shallow configurations in our framework (detailed in Supplementary Table 2) were designed to balance expressive power with computational efficiency for robust IDR function prediction.

CNN for local pattern extraction. CNNs excel at identifying local, position-invariant motifs through convolutional filters. In IDPFunNet’s hybrid path, we implement a two-layer 1D CNN. The first layer reduces the 1024-dimensional ProtT5 embedding to 512 channels, and the second expands it back to 1024 channels, using a kernel size of 3 and padding to preserve sequence length. This compact design is sufficient to capture local biochemical and evolutionary signatures indicative of binding sites without over-parameterization [1].

BiLSTM for long-range context modeling. BiLSTMs are adept at capturing long-range dependencies and bidirectional context in sequences via their gating mechanisms [2]. In both paths of IDPFunNet, we employ a single-layer BiLSTM with 512 hidden units per direction, processing the 1024-dimensional features to output a 1024-dimensional context-aware representation. This configuration effectively models the contextual relationships within IDRs that are crucial for distinguishing flexible linkers and binding regions.

ResMLP for robust functional scoring. ResMLPs enhance standard MLPs with residual connections, mitigating the vanishing gradient problem and facilitating training of deeper networks [3]. Each predictor in IDPFunNet uses a compact ResMLP block with two residual layers, each containing 512 hidden units. Taking the 1024-dimensional integrated features as input, it outputs a single residue-wise score. This shallow residual design provides non-linear transformation capacity for accurate probability estimation while maintaining stability and efficiency.

In summary, the integration of these specifically configured components—a shallow CNN for local motifs, a single-layer BiLSTM for global context, and a compact ResMLP for prediction—forms the efficient yet powerful core of IDPFunNet. This design is informed by their proven success in bioinformatics [4-8] and is optimized for the specific task of multi-functional IDR annotation.

# Supplementary Note 2: Bootstrap resampling process for hypothesis testing

To enable a robust and statistically valid comparison, we applied a bootstrap resampling framework for hypothesis testing on six independent test datasets. The detailed workflow is illustrated in Supplementary Figure 1.

Let *M* and *N* denote the total numbers of positive and negative sequences in a dataset, respectively. In each bootstrap iteration, we generated a subsample of target size *K* = (*M* + *N*)/2. The sampling logic addressed class imbalance as follows:

- Standard sampling (*M* ≥ 10): Subsampling was performed randomly while preserving the original class distribution. The expected numbers of positives (*m*) and negatives (*n*) in the subsample are: $m=K*\frac{M}{M+N}=\frac{M}{2}, n=K*\frac{N}{M+N}=\frac{N}{2}$
- Negative subsampling for scarce positives (*M* < 10, e.g., TE83 lipid-binding): To avoid overfitting and maintain statistical reliability, we retained all *M* positive sequences. We then randomly subsampled negatives to reach the target size *K*: $m=M, n=K-M=\frac{M+N}{2}- M$.

Consequently, the subsampled dataset in each iteration contains *m* positive and *n* negative sequences, where:

$$m=\left\{ \begin{matrix} M, & M<10 \\ \frac{M}{2}, & M\geq10 \end{matrix} \right., n=\frac{M+N}{2}-m$$

This framework explicitly handles severe class imbalance by ensuring all available positive data is utilized when *M* < 10, while the subsample size is consistently controlled across iterations for fair comparison.

# Supplementary Note 3: introduction of ESM-family models and ProtT5

Recent advances in protein language models (PLMs) have significantly expanded computational capabilities across structural biology, functional annotation, and protein design [9-16]. In IDPs’ research, PLMs such as ESM-1/2 (Evolutionary Scale Modeling) [9, 10] and ProtT5 [11] have demonstrated the ability to capture evolutionary and biophysical signatures at the residue level, thereby enhancing predictions of binding motifs and dynamic regions [17, 18].

ESM-1b is a deep transformer encoder model with 650 million parameters, trained on a high-diversity sparse dataset (UR50/S) that includes UniRef50 representative sequences. This model effectively encodes information about biochemical properties, remote homology, and alignments within protein families, making it applicable to downstream tasks such as remote homology detection, linear projections, and the prediction of secondary structure, contact points, and mutational effects [9].

ESM2 employs a BERT-style encoder-only transformer architecture and is trained on approximately 65 million unique sequences. Its primary task is protein structure prediction, where it has demonstrated strong performance [10].

ProtT5, with a parameter count of 3 billion, uses a transformer architecture with an encoder-decoder structure. It captures biophysical features of amino acids, protein structure classes, domains of life and viruses, and protein functions in conserved motifs[11]. ProtT5 has shown outstanding performance in downstream tasks, including the prediction of secondary structure, subcellular location, and protein classification [19-22].

Drawing on these capabilities, we investigated the potential of PLMs (ProtT5, ESM-1b, and ESM2) to encode evolutionary information, functional motifs, and structural characteristics of intrinsically disordered regions (IDRs) involved in six disordered functions. When designing the IDPFunNet model, we utilized the ProtT5 model. This approach leverages the strengths of PLMs to enhance the prediction of IDRs’ functions.

# Supplementary Note 4: AlphaFold2-derived features for sequence encoding

AlphaFold2 (AF2) [23], a deep learning-based framework for end-to-end protein structure prediction, infers tertiary structures from amino acid sequences while generating auxiliary metrics to assess structural reliability and dynamics. These include 8-state secondary structure, torsion angles (ψ and φ) of the peptide backbone, relative solvent accessibility (RSA), quantifying residue exposure to solvent environments, predicted local-distance difference test (pLDDT) scores (0–100, reflecting residue-level structural confidence) and predicted aligned error (PAE, estimating positional uncertainties between residue pairs to infer domain flexibility).

These metrics encode intrinsic disorder dynamics. Low pLDDT (<50) and elevated PAE values correlate strongly with IDRs [24, 25], a principle exploited in CAID challenges through AF2-derived models (e.g., AlphaFold-pLDDT, AlphaFold-rsa) for IDR and its function prediction [26]. Building on this, we encoded each residue in input sequences using five AF2-derived feature types:

1. 8-dimensional one-hot vector: 8-class secondary structure probabilities.
2. 4-dimensional vector: cosin and sin of Ψ/Φ torsion angles normalized to [−π, π].
3. 1-dimensional scalar: RSA value.
4. 1-dimensional scalar: pLDDT confidence score.
5. 32-dimensional histogram: PAE values aggregated into 32 equal-width bins (0–32 Å), computed by binning pairwise errors across the sequence.
6. 3-dimensional vector: sequence position of each residue, as well as its distance to the N- and C-terminus, normalized by the sequence length.

This yielded a 49-dimensional feature vector per residue, enabling systematic evaluation of AF2-derived features for IDR multifunction prediction.

# Supplementary Note 5: predicted DNA and RNA merging protocol

We predict nucleic acid-binding IDRs, which encompass both RNA- and DNA-binding IDRs. For comparative fairness against existing methods—DisoFLAG [18] and DeepDISOBind [27], both capable of predicting RNA- and DNA-binding IDRs—we integrated their binary predictions and propensity scores using the following strategy:

1. The binary prediction ($b_{i}$) for residue *i*:

$$b_{i}=\left\{ \begin{matrix} 1, & b_{i}^{R}+b_{i}^{D}\geq1 \\ 0, & b_{i}^{R}+b_{i}^{D}=0 \end{matrix} \right. ,$$

where $b_{i}^{R}\in\left\{ 0,1 \right\}$ and $b_{i}^{D}\in\left\{ 0,1 \right\}$ denote the binary prediction of disordered RNA- and DNA-binding residues, respectively. The disordered RNA- or DNA-binding residues means the residues located in RNA- or DNA-binding IDRs.

1. The propensity score ($p_{i}$) for residue *i*:

$$p_{i}=\left\{ \begin{matrix} \frac{b_{i}^{R}*p_{i}^{R}+b_{i}^{D}*p_{i}^{D}}{b_{i}^{R}+b_{i}^{D}}, & b_{i}^{R}+b_{i}^{D}\geq1 \\ \frac{p_{i}^{R}+p_{i}^{D}}{2} & b_{i}^{R}+b_{i}^{D}=0 \end{matrix} \right. ,$$

Here, $p_{i}^{R}$​ and $p_{i}^{D}$ represent the propensity scores for likelihoods being the disordered RNA- and DNA-binding residues. The final output ($b_{i}, p_{i}$) thus provides unified binary and probabilistic predictions for disordered nucleic acid-binding residues.

# Supplementary Note 6: Re-training of DisoFLAG and DeepDISOBind for a Fair Comparison

To isolate the influence of training data scale and ensure a fair architectural comparison, we retrained DisoFLAG and DeepDISOBind on our unified datasets (TR552/VA227), denoted as DisoFLAG* and DeepDISOBind*. Training strictly adhered to the original [18, 27], preserving each model’s architecture, loss functions, and hyperparameters (e.g., DisoFLAG’s summed binary cross-entropy over 50 epochs; DeepDISOBind’s uncertainty-weighted loss with native early-stopping).

Performance evaluation on TE210 and TE83 (Supplementary Tables 3-4, Supplementary Figure 6) revealed that retraining yielded only limited and inconsistent gains. DisoFLAG* showed a 0.8–6.8% increase in AUC for protein binding but declined in nucleic acid binding (e.g., on TE210, AUC decreased from 0.864 to 0.823 and APS from 0.181 to 0.107) and DFL prediction (AUC dropped by 1.7–6.5%). DeepDISOBind* improved nucleic acid binding AUC by 4.9% on TE210 but regressed by 7.0% on TE83. Critically, the relative performance ranking between IDPFunNet and the retrained models remained unchanged from the original comparison. IDPFunNet maintained its dominant position in protein- and lipid-binding, and the significant accuracy gaps in its favor persisted.

These results demonstrate that the additional training data provided only marginal, task-specific benefits to the existing architectures. Therefore, IDPFunNet’s superior task-specific performance is primarily attributable to its novel network design, robustly confirming its architectural advantage under equitable training conditions.

# Supplementary Tables

**Supplementary Table 1. Details about the eight datasets of TR552, VA227, TE210, TE83, CAID2_Bind, CAID2_DFL, CAID3_Bind and CAID3_DFL.** The column of “Function type” lists all six types of functions we considered. The binding IDR covers the interaction between IDRs and proteins, nucleic acids, lipids, ions and some other small molecules. The “# positive proteins/IDRs” presents the number of proteins/IDRs annotated with a given function. If a residue is located in the IDR with a given functional annotation, it is defined as “positive”; otherwise, it is regarded as “negative”. Notably, no more than 30 intrinsically disordered proteins (IDPs) are annotated with nucleic acid binding IDRs across VA227, TE210 and TE83. Consequently, to ensure sufficient data for training and testing, this IDR category is not subdivided into DNA binding and RNA binding subtypes.

| **Dataset** | **Function type** | **# positive proteins** | **# positive IDRs** | **# positives** | **# negatives** |
| --- | --- | --- | --- | --- | --- |
| TR552 | Protein binding | 336 | 398 | 35777 | 276296 |
|  | Nucleic acid binding | 73 | 86 | 7702 | 304371 |
|  | Lipid binding | 28 | 32 | 2257 | 309816 |
|  | Ion binding | 34 | 36 | 4732 | 307341 |
|  | Other small molecules binding | 22 | 27 | 1159 | 310914 |
|  | DFL | 124 | 145 | 5385 | 306688 |
| VA227 | Protein binding | 125 | 137 | 12345 | 114270 |
|  | Nucleic acid binding | 30 | 36 | 2761 | 123854 |
|  | Lipid binding | 13 | 13 | 1150 | 125465 |
|  | Ion binding | 10 | 10 | 894 | 125721 |
|  | Other small molecules binding | 8 | 9 | 265 | 126350 |
|  | DFL | 67 | 76 | 3010 | 123605 |
| TE210 | Protein binding | 129 | 142 | 10963 | 86165 |
|  | Nucleic acid binding | 28 | 34 | 2402 | 94726 |
|  | Lipid binding | 12 | 13 | 1530 | 95598 |
|  | Ion binding | 11 | 12 | 760 | 96368 |
|  | Other small molecules binding | 4 | 4 | 722 | 96406 |
|  | DFL | 57 | 64 | 2294 | 94834 |
| TE83 | Protein binding | 50 | 58 | 6473 | 42326 |
|  | Nucleic acid binding | 8 | 8 | 698 | 48101 |
|  | Lipid binding | 6 | 6 | 460 | 48339 |
|  | Ion binding | 6 | 6 | 562 | 48237 |
|  | DFL | 23 | 32 | 920 | 47879 |
| CAID2_Bind | Disordered binding | 78 | 88 | 8209 | 58960 |
| CAID2_DFL | DFL | 40 | 42 | 2023 | 35127 |
| CAID3_Bind | Disordered binding | 51 | 55 | 4038 | 20571 |
| CAID3_DFL | DFL | 20 | 25 | 952 | 13926 |

**Supplementary Table 2. Specification of core architectural modules in IDPFunNet.** This table provides the complete configuration and hyperparameters for the CNN, BiLSTM, and ResMLP components described in the Model Architecture section and Supplementary Note 1. For each module, the input/output dimensions and key parameters (e.g., kernel size, hidden units, layer counts) are detailed.

| **Dual-Path** | **Module** | **Submodule / Operation** | **Hyperparameters**  **/ Description** |
| --- | --- | --- | --- |
| The hybrid CNN- BiLSTM path | CNN | Two Conv1D layers | The first layer: Input = 1024, Output = 512;  The second layer: Input = 512, Output = 1024;  Kernel=3, Pad=1; |
|  |  | Linear | Input = 1024, Output = 512 |
|  | BiLSTM | LSTM | Input = 1024, Hidden = 512, Layers = 1,  Bidirectional = True, Output = 1024 |
|  |  | Linear | Input = 1024, Output = 512 |
|  | ResMLP (x5) | Residual MLP block | Input = 1024, Hidden = 512, Blocks = 2, Output = 1 |
| The pure BiLSTM path | BiLSTM | LSTM | Input = 1024, Hidden = 512, Layers = 1,  Bidirectional = True, Output = 1024 |
|  | ResMLP (x1) | Residual MLP block | Input = 1024, Hidden = 512, Blocks = 2, Output = 1 |

**Supplementary Table 3. Bootstrap-based performance and stability (mean ± std) of predictors on TE210 and TE83.** This table reports the mean and standard deviation of evaluation metrics (AUC, APS, F1-max, MCC) across 30 bootstrap iterations for each prediction category and dataset. These values quantify the central tendency and variability of each method’s performance, serving as the basis for all statistical significance tests. The asterisk (*) denotes models retrained on our benchmark datasets.

| **Dataset** | **prediction** | **method** | **AUC** | **APS** | **F1_max** | **MCC** |
| --- | --- | --- | --- | --- | --- | --- |
| TE210 | Protein binding IDR | **IDPFunNet** | 0.864±0.014 | 0.416±0.060 | 0.473±0.042 | 0.394±0.032 |
|  |  | DisoFLAG* | 0.858±0.015 | 0.412±0.062 | 0.447±0.048 | 0.380±0.037 |
|  |  | DisoFLAG | 0.853±0.017 | 0.378±0.047 | 0.458±0.034 | 0.368±0.028 |
|  |  | DeepDISOBind* | 0.796±0.028 | 0.395±0.093 | 0.406±0.063 | 0.292±0.042 |
|  |  | DeepDISOBind | 0.792±0.027 | 0.357±0.074 | 0.414±0.062 | 0.314±0.055 |
|  |  | DisoLipPred | 0.651±0.023 | 0.163±0.019 | 0.274±0.026 | 0.066±0.028 |
|  | Nucleic acid binding IDR | DisoFLAG | 0.862±0.032 | 0.200±0.060 | 0.285±0.058 | 0.200±0.030 |
|  |  | DeepDISOBind* | 0.822±0.053 | 0.225±0.075 | 0.366±0.067 | 0.195±0.041 |
|  |  | DisoFLAG* | 0.824±0.032 | 0.116±0.039 | 0.190±0.053 | 0.161±0.035 |
|  |  | DeepDISOBind | 0.784±0.043 | 0.135±0.056 | 0.223±0.063 | 0.172±0.043 |
|  |  | **IDPFunNet** | 0.771±0.032 | 0.064±0.016 | 0.129±0.031 | 0.098±0.037 |
|  |  | DisoLipPred | 0.620±0.037 | 0.037±0.007 | 0.080±0.015 | 0.021±0.017 |
|  | Lipid binding IDR | **IDPFunNet** | 0.894±0.061 | 0.428±0.236 | 0.461±0.178 | 0.421±0.171 |
|  |  | DisoFLAG* | 0.877±0.059 | 0.228±0.152 | 0.307±0.147 | 0.246±0.146 |
|  |  | DisoFLAG | 0.865±0.054 | 0.267±0.170 | 0.308±0.143 | 0.161±0.037 |
|  |  | DeepDISOBind*-PB | 0.793±0.079 | 0.094±0.063 | 0.166±0.078 | 0.136±0.046 |
|  |  | DeepDISOBind-PB | 0.786±0.083 | 0.080±0.053 | 0.158±0.082 | 0.142±0.065 |
|  |  | DisoLipPred | 0.668±0.077 | 0.031±0.010 | 0.071±0.023 | 0.058±0.045 |
|  | Ion binding IDR | DeepDISOBind-NB | 0.777±0.062 | 0.036±0.020 | 0.100±0.047 | 0.068±0.040 |
|  |  | **IDPFunNet** | 0.740±0.063 | 0.015±0.005 | 0.036±0.012 | -0.008±0.002 |
|  |  | DeepDISOBind*-PB | 0.738±0.063 | 0.017±0.008 | 0.049±0.022 | 0.076±0.040 |
|  |  | DisoFLAG | 0.724±0.063 | 0.016±0.005 | 0.041±0.012 | 0.072±0.016 |
|  |  | DisoLipPred | 0.643±0.048 | 0.012±0.003 | 0.029±0.007 | 0.010±0.021 |
|  |  | DisoFLAG* | 0.638±0.126 | 0.016±0.008 | 0.048±0.025 | 0.009±0.021 |
|  | Other small molecule binding IDR | DisoFLAG*-LB | 0.906±0.005 | 0.318±0.047 | 0.374±0.031 | 0.337±0.031 |
|  |  | **IDPFunNet** | 0.871±0.009 | 0.436±0.026 | 0.485±0.033 | 0.131±0.009 |
|  |  | DisoFLAG-LB | 0.871±0.006 | 0.363±0.043 | 0.421±0.028 | 0.148±0.006 |
|  |  | DeepDISOBind-PB | 0.842±0.016 | 0.084±0.025 | 0.188±0.045 | 0.159±0.014 |
|  |  | DeepDISOBind*-PB | 0.838±0.017 | 0.084±0.033 | 0.167±0.046 | 0.145±0.011 |
|  |  | DisoLipPred | 0.657±0.009 | 0.021±0.001 | 0.050±0.002 | -0.014±0.002 |
|  | DFL | DisoFLAG | 0.901±0.017 | 0.290±0.053 | 0.410±0.052 | 0.315±0.033 |
|  |  | **IDPFunNet** | 0.882±0.015 | 0.231±0.045 | 0.339±0.044 | 0.329±0.041 |
|  |  | DisoFLAG* | 0.841±0.020 | 0.140±0.035 | 0.222±0.036 | 0.203±0.039 |
| TE83 | Protein binding IDR | **IDPFunNet** | 0.826±0.030 | 0.433±0.087 | 0.464±0.058 | 0.346±0.053 |
|  |  | DisoFLAG* | 0.814±0.026 | 0.333±0.060 | 0.466±0.054 | 0.381±0.046 |
|  |  | DisoFLAG | 0.761±0.042 | 0.270±0.048 | 0.392±0.053 | 0.269±0.053 |
|  |  | DeepDISOBind* | 0.754±0.049 | 0.335±0.098 | 0.410±0.072 | 0.289±0.066 |
|  |  | DeepDISOBind | 0.752±0.049 | 0.340±0.098 | 0.389±0.069 | 0.257±0.080 |
|  |  | DisoLipPred | 0.601±0.034 | 0.170±0.034 | 0.287±0.044 | 0.022±0.029 |
|  | Nucleic acid binding IDR | **IDPFunNet** | 0.734±0.131 | 0.050±0.038 | 0.123±0.081 | 0.118±0.098 |
|  |  | DisoFLAG* | 0.716±0.136 | 0.052±0.035 | 0.109±0.057 | 0.080±0.058 |
|  |  | DeepDISOBind | 0.691±0.148 | 0.034±0.016 | 0.090±0.038 | 0.097±0.052 |
|  |  | DisoFLAG | 0.655±0.200 | 0.109±0.058 | 0.200±0.078 | 0.091±0.062 |
|  |  | DeepDISOBind* | 0.651±0.122 | 0.036±0.017 | 0.092±0.032 | 0.078±0.046 |
|  |  | DisoLipPred | 0.525±0.076 | 0.017±0.007 | 0.039±0.016 | -0.004±0.016 |
|  | Lipid binding IDR | DisoFLAG | 0.944±0.008 | 0.483±0.061 | 0.528±0.054 | 0.235±0.010 |
|  |  | DeepDISOBind*-NB | 0.845±0.017 | 0.057±0.007 | 0.128±0.014 | 0.211±0.012 |
|  |  | **IDPFunNet** | 0.843±0.015 | 0.356±0.096 | 0.477±0.089 | 0.304±0.052 |
|  |  | DisoFLAG* | 0.824±0.016 | 0.439±0.043 | 0.532±0.047 | 0.407±0.068 |
|  |  | DeepDISOBind-NB | 0.815±0.016 | 0.176±0.050 | 0.291±0.028 | 0.138±0.013 |
|  |  | DisoLipPred | 0.561±0.016 | 0.022±0.002 | 0.047±0.005 | -0.013±0.004 |
|  | Ion binding IDR | DisoFLAG* | 0.814±0.021 | 0.249±0.124 | 0.381±0.095 | 0.348±0.091 |
|  |  | DisoFLAG | 0.803±0.019 | 0.246±0.120 | 0.398±0.068 | 0.110±0.014 |
|  |  | **IDPFunNet** | 0.771±0.018 | 0.295±0.103 | 0.399±0.097 | 0.353±0.109 |
|  |  | DeepDISOBind-PB | 0.657±0.030 | 0.044±0.010 | 0.116±0.031 | 0.095±0.019 |
|  |  | DeepDISOBind*-NB | 0.613±0.024 | 0.064±0.007 | 0.173±0.015 | 0.066±0.012 |
|  |  | DisoLipPred | 0.577±0.020 | 0.028±0.003 | 0.060±0.005 | -0.003±0.005 |
|  | DFL | **IDPFunNet** | 0.788±0.038 | 0.089±0.032 | 0.153±0.044 | 0.127±0.052 |
|  |  | DisoFLAG | 0.757±0.056 | 0.082±0.026 | 0.163±0.044 | 0.123±0.043 |
|  |  | DisoFLAG* | 0.742±0.038 | 0.091±0.054 | 0.163±0.070 | 0.090±0.052 |

**Supplementary Table 4. Comparative performance summary and statistical significance testing on TE210 and TE83.** This table provides a synthesized comparison between IDPFunNet and state-of-the-art methods. The best-performing value per category is highlighted in bold. Symbols “+”, “−”, and “=” adjacent to a comparator’s metric indicate that it is statistically superior, inferior, or not significantly different (p < 0.05) from IDPFunNet, respectively, based on hypothesis testing of the bootstrap distributions in Supplementary Table 3. The asterisk (*) denotes retrained models.

| **Dataset** | **Prediction** | **method** | **AUC** | **APS** | **F1_max** | **MCC** |
| --- | --- | --- | --- | --- | --- | --- |
| TE210 | Protein binding IDR | **IDPFunNet** | **0.866** | **0.420** | **0.477** | **0.399** |
|  |  | DisoFLAG* | 0.860- | 0.410= | 0.445- | 0.383- |
|  |  | DisoFLAG | 0.853- | 0.370- | 0.455- | 0.371- |
|  |  | DeepDISOBind* | 0.802- | 0.416= | 0.410- | 0.299- |
|  |  | DeepDISOBind | 0.797- | 0.368- | 0.418- | 0.318- |
|  |  | DisoLipPred | 0.651- | 0.164- | 0.274- | 0.067- |
|  | Nucleic acid binding IDR | DisoFLAG | **0.864+** | **0.181+** | **0.270+** | **0.200+** |
|  |  | DeepDISOBind* | 0.829+ | 0.224+ | 0.371+ | 0.193+ |
|  |  | DisoFLAG* | 0.823+ | 0.107+ | 0.182+ | 0.160+ |
|  |  | DeepDISOBind | 0.790+ | 0.119+ | 0.222+ | 0.172+ |
|  |  | **IDPFunNet** | 0.775 | 0.063 | 0.123 | 0.103 |
|  |  | DisoLipPred | 0.628- | 0.036- | 0.078- | 0.023- |
|  | Lipid binding IDR | **IDPFunNet** | **0.896** | **0.436** | **0.454** | **0.430** |
|  |  | DisoFLAG* | 0.879- | 0.204- | 0.312- | 0.258- |
|  |  | DisoFLAG | 0.865- | 0.246- | 0.305- | 0.156- |
|  |  | DeepDISOBind*-PB | 0.788- | 0.068- | 0.139- | 0.129- |
|  |  | DeepDISOBind-PB | 0.782- | 0.056- | 0.135- | 0.132- |
|  |  | DisoLipPred | 0.662- | 0.028- | 0.066- | 0.059- |
|  | Ion binding IDR | DeepDISOBind-NB | **0.780+** | **0.027+** | **0.072+** | 0.066+ |
|  |  | **IDPFunNet** | 0.748 | 0.015 | 0.035 | -0.008 |
|  |  | DeepDISOBind*-PB | 0.746= | 0.016= | 0.046+ | **0.075+** |
|  |  | DisoFLAG | 0.732- | 0.015= | 0.035= | 0.073+ |
|  |  | DisoLipPred | 0.644- | 0.011- | 0.025- | 0.007+ |
|  |  | DisoFLAG* | 0.626- | 0.013= | 0.036= | 0.012+ |
|  | Other small molecule binding IDR | DisoFLAG*-LB | **0.905+** | 0.206- | 0.263- | **0.254+** |
|  |  | **IDPFunNet** | 0.870 | **0.354** | **0.391** | 0.093 |
|  |  | DisoFLAG-LB | 0.869= | 0.269- | 0.355- | 0.105+ |
|  |  | DeepDISOBind-PB | 0.840- | 0.038- | 0.109- | 0.113+ |
|  |  | DeepDISOBind*-PB | 0.836- | 0.035- | 0.090- | 0.103+ |
|  |  | DisoLipPred | 0.656- | 0.010- | 0.026- | -0.010- |
|  | DFL | DisoFLAG | **0.903+** | **0.282+** | **0.404+** | 0.313- |
|  |  | **IDPFunNet** | 0.883 | 0.227 | 0.336 | **0.329** |
|  |  | DisoFLAG* | 0.844- | 0.133- | 0.219- | 0.210- |
| TE83 | Protein binding IDR | **IDPFunNet** | **0.832** | **0.427** | 0.450 | 0.355 |
|  |  | DisoFLAG* | 0.822- | 0.330- | **0.467=** | **0.391+** |
|  |  | DisoFLAG | 0.770- | 0.273- | 0.388- | 0.283- |
|  |  | DeepDISOBind* | 0.769- | 0.333- | 0.406- | 0.302- |
|  |  | DeepDISOBind | 0.764- | 0.337- | 0.384- | 0.274- |
|  |  | DisoLipPred | 0.599- | 0.162- | 0.281- | 0.018- |
|  | Nucleic acid binding IDR | **IDPFunNet** | **0.743** | **0.037** | **0.107** | **0.116** |
|  |  | DisoFLAG* | 0.717- | 0.035= | 0.084= | 0.077- |
|  |  | DeepDISOBind | 0.701- | 0.031- | 0.085- | 0.101= |
|  |  | DisoFLAG | 0.665- | 0.086+ | 0.191+ | 0.096- |
|  |  | DeepDISOBind* | 0.652- | 0.031- | 0.081- | 0.076- |
|  |  | DisoLipPred | 0.537- | 0.015- | 0.032- | -0.002- |
|  | Lipid binding IDR | DisoFLAG | **0.948+** | **0.369+** | **0.443+** | 0.172- |
|  |  | DeepDISOBind*-NB | 0.851= | 0.037- | 0.070- | 0.152- |
|  |  | **IDPFunNet** | 0.848 | 0.213 | 0.351 | 0.217 |
|  |  | DisoFLAG* | 0.827- | 0.363+ | 0.461+ | **0.295+** |
|  |  | DeepDISOBind-NB | 0.823- | 0.097- | 0.249- | 0.105- |
|  |  | DisoLipPred | 0.562- | 0.011- | 0.025- | -0.010- |
|  | Ion binding IDR | DisoFLAG* | **0.818+** | 0.113- | 0.262- | 0.244= |
|  |  | DisoFLAG | 0.807+ | 0.117- | **0.294=** | 0.078- |
|  |  | **IDPFunNet** | 0.774 | **0.170** | 0.276 | **0.248** |
|  |  | DeepDISOBind-PB | 0.660- | 0.020- | 0.062- | 0.066- |
|  |  | DeepDISOBind*-NB | 0.620- | 0.031- | 0.109- | 0.047- |
|  |  | DisoLipPred | 0.577- | 0.013- | 0.028- | -0.002- |
|  | DFL | **IDPFunNet** | **0.791** | **0.087** | 0.151 | **0.135** |
|  |  | DisoFLAG | 0.764- | 0.087= | **0.164=** | 0.127= |
|  |  | DisoFLAG* | 0.751- | 0.073= | 0.160= | 0.100- |

**Supplementary Table 5.** **Comparison of predictive performance between IDPFunNet and top methods from CAID2/3 challenges, using AUC, APS, F1-max, and MCC metrics.** IDPFunNet components for DFL and protein-binding IDR prediction were utilized for the assessment of DFL and generic binding IDR, respectively. The model variants DisoFLAG-LB/IB/PB/DFL denote task-specific components of the DisoFLAG framework, where PB/LB/IB is the abbreviation of protein/lipid/ion binding. The top-ranked DisoFLAG_IDR (CAID3) included in CAID2/3_DFL benchmarking despite its IDR specialization. The “Note” column records the top five AUC-ranked predictors in CAID2/3 challenges. Following the strategy in CAID2 challenge, coverage is applied to quantify the fraction of predicted targets generated by each method [26]. A "+"/"−" symbol next to a metric value indicates that the corresponding method is significantly better/worse than IDPFunNet (p-value < 0.05). It is important to note that statistical hypothesis testing on CAID3_DFL was conducted on its subset of 14 proteins predicted by all compared methods. This led to inconsistencies between the statistical test and the overall performance on the entire set CAID3_DFL. Consequently, this table does not include the statistical significance on CAID3_DFL.

| **Dataset** | **Method** | **AUC** | **APS** | **F1-max** | **MCC** | **Coverage** | **Note** |
| --- | --- | --- | --- | --- | --- | --- | --- |
| CAID2_Bind | DisoFLAG-PB | **0.879+** | **0.563+** | **0.554+** | **0.489+** | 1 |  |
|  | **IDPFunNet** | 0.861 | 0.466 | 0.497 | 0.42 | 1 |  |
|  | DisoFLAG-LB | 0.847- | 0.426= | 0.511+ | 0.436+ | 1 |  |
|  | DisoFLAG-IB | 0.84- | 0.498+ | 0.495= | 0.418= | 1 |  |
|  | ENSHROUD-protein | 0.753- | 0.252- | 0.361- | 0.275- | 1 | Top 1 |
|  | ENSHROUD-all | 0.752- | 0.234- | 0.36- | 0.276- | 1 | Top 2 |
|  | MoRFchibi-Web | 0.751- | 0.284- | 0.355- | 0.259- | 1 | Top 3 |
|  | DeepDRPBind-protein | 0.746- | 0.234- | 0.372- | 0.267- | 0.987 | Top 4 |
|  | ENSHROUD-nucleic | 0.743- | 0.233- | 0.351- | 0.259- | 1 | Top 5 |
| CAID2_DFL | **IDPFunNet** | **0.825** | **0.198** | 0.283 | 0.255 | 1 |  |
|  | DisoFLAG-IDR | 0.821- | 0.182- | 0.29= | 0.258= | 1 |  |
|  | DisoFLAG-DFL | 0.8- | 0.197- | 0.258- | 0.23- | 1 |  |
|  | SPOT-Disorder2 | 0.782- | 0.153- | **0.292**+ | **0.276**+ | 0.775 | Top 1 |
|  | AlphaFold-rsa | 0.77- | 0.103- | 0.225- | 0.245- | 0.925 | Top 2 |
|  | SETH-0 | 0.77- | 0.157- | 0.22- | 0.215- | 1 | Top 3 |
|  | SETH-1 | 0.762- | 0.133- | 0.229- | 0.185- | 1 | Top 4 |
|  | Dispredict3 | 0.744- | 0.148- | 0.275- | 0.239- | 1 | Top 5 |
| CAID3_Bind | **IDPFunNet** | **0.892** | 0.66 | **0.616** | **0.537** | 1 |  |
|  | DisoFLAG-LB | 0.862- | **0.666**= | 0.572- | 0.479- | 0.98 | Top 1 |
|  | IPA-All-bind | 0.856- | 0.552- | 0.589- | 0.528= | 1 | Top 2 |
|  | IPA-Nucleotide-bind | 0.851- | 0.534- | 0.53- | 0.43- | 1 | Top 3 |
|  | IPA-Protein-bind | 0.85- | 0.518- | 0.545- | 0.457- | 1 | Top 4 |
|  | DisoFLAG-IB | 0.832- | 0.619- | 0.551- | 0.445- | 0.98 | Top 5 |
|  | DisoFLAG-PB | 0.776- | 0.376- | 0.48- | 0.347- | 0.98 |  |
| CAID3_DFL | IPA-AF2-Linker | **0.881** | 0.377 | 0.456 | 0.441 | 0.9 | Top 1 |
|  | DisoFLAG-IDR | 0.862 | 0.327 | 0.45 | 0.425 | 0.95 | Top 2 |
|  | LINKER-Pred2 | 0.854 | 0.359 | 0.485 | 0.455 | 1 | Top 3 |
|  | DisorderUnetLM | 0.851 | 0.258 | 0.374 | 0.356 | 1 | Top 4 |
|  | ESMDisPred-2 | 0.85 | 0.366 | **0.531** | **0.463** | 0.7 | Top 5 |
|  | **IDPFunNet** | 0.839 | **0.427** | 0.462 | 0.429 | 1 |  |
|  | DisoFLAG-DFL | 0.822 | 0.366 | 0.378 | 0.348 | 0.95 |  |

# Supplementary Figures

**Supplementary Figure 1.** Bootstrap resampling workflow for hypothesis testing. The schematic illustrates the process of constructing balanced subsets for statistical comparison. Dataset refers to one of the six independent test sets, containing *M* positive sequences (PSEQ) and *N* negative sequences (NSEQ). Through bootstrap resampling, 30 subsets (S_1_, S_2_, …, S_30_) are generated. Each subset S*_i_* contains *m* PSEQ and *n* NSEQ, sampled according to the strategy defined in Supplementary Note 2 to address class imbalance.

**Supplementary Figure 2. (a)** ROC and **(b)** PRE-REC curves for IDPFunNet and SOTA methods on the test set TE210. PB/NB/LB/IB/SB denote the IDRs binding to protein/nucleic acid/lipid/ion/other small molecules. The model variants DeepDISOBind-PB, DeepDISOBind-NB and DisoFLAG-LB correspond to task-specific components of DeepDISOBind and DisoFLAG frameworks

**Supplementary Figure 3. (a)** ROC and **(b)** PRE-REC curves for IDPFunNet and SOTA methods on the test set TE83. PB/NB/LB/IB/SB denote the IDRs binding to protein/nucleic acid/lipid/ion/other small molecules. The model variants DeepDISOBind-PB, DeepDISOBind-NB and DisoFLAG-LB correspond to task-specific components of DeepDISOBind and DisoFLAG frameworks. Notably, small molecule-binding IDR annotations are unavailable in TE83.

**Supplementary Figure 4.** **(a)** ROC and **(b)** PRE-REC curves for IDPFunNet and SOTA methods on the test set CAID2_Bind; **(c)** and **(d)** on the test set CAID2_DFL. The model variants DisoFLAG-PB/-LB/-IB correspond to task-specific components of DisoFLAG framework, where PB/LB/IB denote protein/lipid/ion binding.

**Supplementary Figure 5. (a)** ROC and **(b)** PRE-REC curves for IDPFunNet and SOTA methods on the test set CAID3_Bind; **(c)** and **(d)** on the test set CAID3_DFL_14. The model variants DisoFLAG-PB/-LB/-IB correspond to task-specific components of DisoFLAG framework, where PB/LB/IB denote protein/lipid/ion binding. Notably, CAID3_DFL_14 comprises 14 sequences extracted from CAID3_DFL, which can be fully predicted by all compared methods.

**Supplementary Figure 6:** Comparative performance of IDPFunNet, original and re-trained DisoFLAG/DeepDISOBind models on independent test sets TE210 (**a** and **b**) and TE83 (**c** and **d**), as measured by AUC (**a** and **c**) and APS (**b** and **d**). "+"/"−" next to a given AUC and APS value indicates that the corresponding method is significantly better/worse than IDPFunNet (p-value < 0.05), based on hypothesis testing of the bootstrap distributions in Supplementary Table 3. The asterisk (*) denotes models retrained on our benchmark datasets. IDPFunNet and DisoFLAG / DisoFLAG* support the prediction of protein-, nucleic acid-, lipid-, and ion-binding IDRs and DFLs, whereas DeepDISOBind / DeepDISOBind* are designed to predict protein- and nucleic acid-binding IDRs.

# Supplementary References

1. Krizhevsky A, Sutskever I, Hinton GE. ImageNet Classification with Deep Convolutional Neural Networks, Communications of the Acm 2017;60:84-90.

2. Huang Z, Xu W, Yu K. Bidirectional LSTM-CRF Models for Sequence Tagging, arXiv e-prints 2015:arXiv:1508.01991.

3. Touvron H, Bojanowski P, Caron M et al. ResMLP: Feedforward networks for image classification with data-efficient training, arXiv e-prints 2021:arXiv:2105.03404.

4. Kulmanov M, Hoehndorf R. DeepGOPlus: improved protein function prediction from sequence, Bioinformatics 2020;36:422-429.

5. Dhanuka R, Singh JP, Tripathi A. A Comprehensive Survey of Deep Learning Techniques in Protein Function Prediction, Ieee-Acm Transactions on Computational Biology and Bioinformatics 2023;20:2291-2301.

6. Zhu YH, Zhang CX, Yu DJ et al. Integrating unsupervised language model with triplet neural networks for protein gene ontology prediction, Plos Computational Biology 2022;18.

7. Ke J, Zhao J, Li H et al. Prediction of protein N-terminal acetylation modification sites based on CNN-BiLSTM-attention model, Comput Biol Med 2024;174:108330.

8. Fan Z, Xu Y. Predicting the Functional Changes in Protein Mutations Through the Application of BiLSTM and the Self-Attention Mechanism, Annals of Data Science 2024;11:1077-1094.

9. Rives A, Meier J, Sercu T et al. Biological structure and function emerge from scaling unsupervised learning to 250 million protein sequences, Proc Natl Acad Sci U S A 2021;118.

10. Lin Z, Akin H, Rao R et al. Evolutionary-scale prediction of atomic-level protein structure with a language model, Science 2023;379:1123-1130.

11. Elnaggar A, Heinzinger M, Dallago C et al. ProtTrans: Toward Understanding the Language of Life Through Self-Supervised Learning, IEEE Trans Pattern Anal Mach Intell 2022;44:7112-7127.

12. Brandes N, Ofer D, Peleg Y et al. ProteinBERT: a universal deep-learning model of protein sequence and function, Bioinformatics 2022;38:2102-2110.

13. Ferruz N, Heinzinger M, Akdel M et al. From sequence to function through structure: Deep learning for protein design, Computational Structural Biotechnology Journal 2023;21:238-250.

14. Hou Z, Yang Y, Ma Z et al. Learning the protein language of proteome-wide protein-protein binding sites via explainable ensemble deep learning, Commun Biol 2023;6:73.

15. Kulmanov M, Guzmán-Vega FJ, Duek Roggli P et al. Protein function prediction as approximate semantic entailment, Nature Machine Intelligence 2024;6:220-228.

16. Nijkamp E, Ruffolo JA, Weinstein EN et al. Progen2: exploring the boundaries of protein language models, Cell systems 2023;14:968-978. e963.

17. Xu S, Onoda A. Accurate and fast prediction of intrinsically disordered protein by multiple protein language models and ensemble learning, Journal of Chemical Information Modeling 2023;64:2901-2911.

18. Pang Y, Liu B. DisoFLAG: accurate prediction of protein intrinsic disorder and its functions using graph-based interaction protein language model, BMC Biology 2024;22.

19. Littmann M, Heinzinger M, Dallago C et al. Protein embeddings and deep learning predict binding residues for various ligand classes, Sci Rep 2021;11:23916.

20. Marquet C, Heinzinger M, Olenyi T et al. Embeddings from protein language models predict conservation and variant effects, Hum Genet 2022;141:1629-1647.

21. Stark H, Dallago C, Heinzinger M et al. Light attention predicts protein location from the language of life, Bioinform Adv 2021;1:vbab035.

22. Weissenow K, Heinzinger M, Rost B. Protein language-model embeddings for fast, accurate, and alignment-free protein structure prediction, Structure 2022;30:1169-1177 e1164.

23. Jumper J, Evans R, Pritzel A et al. Highly accurate protein structure prediction with AlphaFold, Nature 2021;596:583-589.

24. Guo HB, Perminov A, Bekele S et al. AlphaFold2 models indicate that protein sequence determines both structure and dynamics, Sci Rep 2022;12:10696.

25. Piovesan D, Monzon AM, Tosatto SCE. Intrinsic protein disorder and conditional folding in AlphaFoldDB, Protein Sci 2022;31:e4466.

26. Del Conte A, Mehdiabadi M, Bouhraoua A et al. Critical assessment of protein intrinsic disorder prediction (CAID) - Results of round 2, Proteins-Structure Function and Bioinformatics 2023;91:1925-1934.

27. Zhang F, Zhao B, Shi W et al. DeepDISOBind: accurate prediction of RNA-, DNA- and protein-binding intrinsically disordered residues with deep multi-task learning, Brief Bioinform 2022;23.
